# Phase Change Material Evolution in Thermal Energy Storage Systems for the Building Sector, with a Focus on Ground-Coupled Heat Pumps

**DOI:** 10.3390/polym14030620

**Published:** 2022-02-05

**Authors:** Silvia Barbi, Francesco Barbieri, Simona Marinelli, Bianca Rimini, Sebastiano Merchiori, Michele Bottarelli, Monia Montorsi

**Affiliations:** 1 Department of Sciences and Methods for Engineering, University of Modena and Reggio Emilia, Via Amendola 2, 42122 Reggio Emilia, Italy; silvia.barbi@unimore.it (S.B.); simona.marinelli@unimore.it (S.M.); bianca.rimini@unimore.it (B.R.); monia.montorsi@unimore.it (M.M.); 2 Interdepartmental Research Center for Industrial Research and Technology Transfer in the Field of Integrated Technologies for Sustainable Research, Efficient Energy Conversion, Energy Efficiency of Buildings, Lighting and Home Automation, EN & TECH, University of Modena and Reggio Emilia, Via Amendola 2, 42122 Reggio Emilia, Italy; 3 TekneHub Laboratory, University of Ferrara, Via Saragat 13, 44122 Ferrara, Italy; sebastiano.merchiori@unife.it (S.M.); michele.bottarelli@unife.it (M.B.); 4 Department of Architecture, University of Ferrara, Via Quartieri 8, 44121 Ferrara, Italy; 5 Interdepartmental Center for Applied Research and Services in Advanced Mechanics and Motoring, INTERMECH-Mo.Re., University of Modena and Reggio Emilia, Via P. Vivarelli 10/1, 41125 Modena, Italy

**Keywords:** phase change materials, latent thermal energy storage, sustainable buildings, ground-coupled heat pumps, energy reduction, materials design, eco-friendly materials, sustainable materials, green economy

## Abstract

The building sector is responsible for a third of the global energy consumption and a quarter of greenhouse gas emissions. Phase change materials (PCMs) have shown high potential for latent thermal energy storage (LTES) through their integration in building materials, with the aim of enhancing the efficient use of energy. Although research on PCMs began decades ago, this technology is still far from being widespread. This work analyses the main contributions to the employment of PCMs in the building sector, to better understand the motivations behind the restricted employment of PCM-based LTES technologies. The main research and review studies are critically discussed, focusing on: strategies used to regulate indoor thermal conditions, the variation of mechanical properties in PCMs-based mortars and cements, and applications with ground-coupled heat pumps. The employment of materials obtained from wastes and natural sources was also taken in account as a possible key to developing composite materials with good performance and sustainability at the same time. As a result, the integration of PCMs in LTES is still in its early stages, but reveals high potential for employment in the building sector, thanks to the continuous design improvement and optimization driven by high-performance materials and a new way of coupling with tailored envelopes.

## 1. Introduction

Since the last decade of the twentieth century, the problem of fossil fuel consumption and the dependency on non-renewable energy sources emerged in the building sector. In this field of application, human beings are facing different challenges: the reduction of consumption, the rise of efficiency in energy production and use, and, lastly, the employment of sustainable resources. In fact, the building sector requires more than 33% of the global energy demand and represents the source of approximately 25% of greenhouse gas emissions [1]. Despite a flattening of CO_2_ emissions between 2013 and 2016, building-related emissions began to grow back in recent years, leading to a total increase equal to 5% from 2010 to 2019. Fossil fuel use has also grown, at an average rate of 0.7%/year, in the same period [2]. The reduction of greenhouse gas emissions and the rational use of energy have become a global widespread endeavor, especially in the European Union (EU), where a relevant need for energy saving in buildings has been recognized and put into practice. In fact, the more recent EU environmental policies for the building sector have promoted the spread of renewable energy technologies [3,4]. The need to enhance sustainable energy employment in this field could be satisfied through different approaches and technologies. 

**Figure 1:**
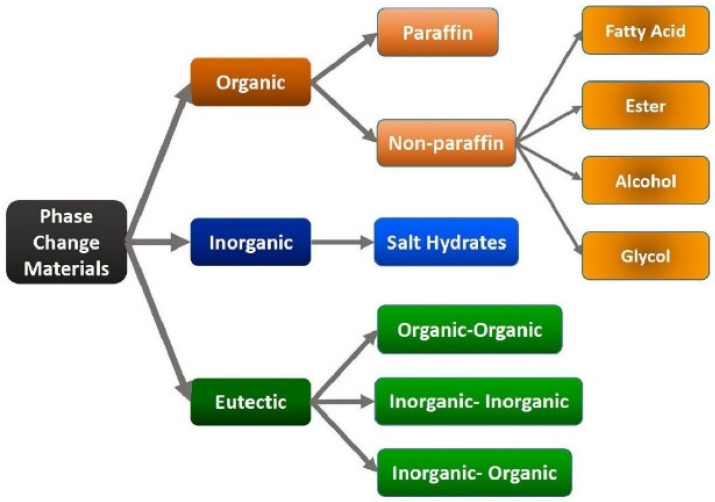
Main classes of PCM [9].

One of these technologies is thermal energy storage (TES), which allows the storage of heat or cool energy in well-tailored materials during low demand periods to release them when needed. The storage mechanism in TES architectures is usually carried out using the sensible or latent heat of specific materials, taking advantage of the specific heat and temperature change with the former, and of the phase-change latent heat performed at a given temperature with the latter [5,6]. Sensible heat TES (SHTES) architectures generally use cheap storage materials, such as water, bricks, or rocks, and for this reason, they are established and widespread technologies. Latent heat TES (LHTES) systems, by contrast, are based on phase change materials (PCMs) and offer the advantages of a fairly constant working temperature and the enhanced energy density of their storage material, which allows the storing of 5–14 times more energy than SHTES in the same volume, therefore reducing the size of the storage system [7,8]. In fact, PCMs can be organic, inorganic, and eutectic compounds characterized by high values of phase-change latent heat. The main classes of PCM are shown in Figure 1.

The majority of organic PCMs are composed of paraffin waxes (CH_3_(CH_2_)_n_CH_3_), with melting temperatures between 20 °C and 70 °C, thereby fulfilling the needs of LHTES architectures in the buildings sector in a wide range of latitudes [6]. Fatty acids (CH_3_(CH_2_)_n_COOH) constitute the second main group of organic PCMs, with melting temperatures between 17 °C and 64 °C. Other organic substances generally exploited as PCMs are esters, alcohols, and glycols [6,10]. Among inorganic PCMs, hydrated salts are the main category. With respect to organic compounds, they have a wider range of melting points, reaching temperatures up to 140 °C [11,12]. Molten salts have also been considered, but their high melting temperatures (>250 °C) make them unattractive for the building sector [13]. Finally, eutectics can also be considered, as they are made of mixtures of two or more pure PCMs, which can be organic–organic, inorganic–inorganic, or inorganic–organic. They show well-defined melting points, but their properties and performances must be further investigated as they are the result of very specific formulations that could be difficult to control for industrial purposes and when handling large volumes of material [9,14]. 

Every category of PCM, taking into account the solid-liquid latent heat, presents several advantages but also drawbacks, as listed in Table 1, which limit their practical and commercial applications in the building sector [15]. There are also some limitations common to all categories, namely high costs, low thermal conductivity, and long-term stability, which make SHTES even more widespread and profitable than LHTES [16]. Among phase changes, solid–liquid is the most employed in practice. In fact, even if the latent heat of the liquid–gas transition is generally higher, the relevant associated volume change must be considered, as it can lead to many severe critical issues in the design of liquid–gas-based TES, such as partially flexible PCM containment or safety devices due to slight temperature changes [6]. On the other hand, PCMs based on solid–solid transition take advantage of the passage between a crystalline and an amorphous solid phase. The lack of a liquid or gas phase prevents leakage problems, but this PCM category shows a lower phase change energy compared to PCMs based on solid–liquid transition. Moreover, their phase change temperatures are usually higher than those required for building applications [9,17].

Since the early studies from 1990 onwards, the use of LHTES technology was considered in the building sector to enhance energy management and consumption, dividing the final PCM applications (for heating or cooling purposes) into active and passive [6]. The significant difference lies in the fact that in active applications, the thermal energy is available only when necessary, using mainly on/off auxiliaries such as heating, ventilation, and air conditioning systems (HVAC). Passive applications, instead, aim to include PCMs directly into building elements, using only the surrounding environment as their energy source, allowing automatic and continuous energy storage or release and providing thermal comfort with minimum or no use of HVAC systems [18,19]. 

Active applications include the use of TES coupled with conventional HVAC systems (e.g., floor or ceiling heating plants) or with heat pumps to optimize the electric energy demand from high-peak to off-peak periods [20]. Other active applications consider solar energy systems and solar air collectors integrated into building walls, as well as the use of ventilation mechanisms with free cooling, that take advantage of low night temperatures to store coldness in building materials and release it during the day. 

Passive technologies include standard solar walls, so-called Trombe walls, and the inclusion of PCMs in construction materials such as concrete, mortars, and insulation materials, or in building elements, such as windows, shutters, wallboards, and bricks [20]. 

An important aspect in all the applications is that the employed PCM must be tailored for a specific use, considering its nature (organic or inorganic), its percentage in the formulation, and, especially, its precise melting temperature according to climatic conditions, building design, and thermal comfort requirements. As PCMs can be fully integrated with the TES architecture, the environmental sustainability of all the system must also be taken into consideration. Several environmental analyses based on the life cycle assessment (LCA) methodology have shown that the environmental impact resulting from the production, installation, and disposal of PCMs is largely recovered from the environmental benefit obtained thanks to energy savings (from 15% to 35% of energy saved based on climatic conditions) [21]. However, other studies have shown that the use of PCMs as paraffins or salt hydrates, while reducing energy consumption, does not significantly reduce the overall impact compared to the use of a conventional insulation material, such as polyurethane, suggesting that the production and the disposal phases of PCM life cycles need to be further investigated [22]. For example, paraffin production is mainly based on fossil oils, although materials derived from renewable sources have also recently been developed [23]. The production process is very similar for all fossil-based materials, regardless of the length of the carbon chain that determines the melting point. The environmental impact can therefore be considered identical for the individual materials [24]. Thereafter, more recent studies focus on more sustainable organic materials not based on fossil fuels. For example, the biodegradability of a plant-based PCM was analyzed and compared to paraffins and salt hydrates, demonstrating the total decomposition of the plant-based material after 4 months [25]. 

Despite the considerable number of studies on PCM integration in LHTES systems, elements, or materials, their application in the building sector is far from being widespread. The aim of this work is to analyze the developments of PCM applications in the building sector, since their early stages, through an analysis of research and review papers. In the first period (2000–2010), described in the Section 2, the main focus was on the improvement of PCMs’ general properties. Next, a focus on the more recent and peculiar applications in the building sector is presented, according to the materials’ evolution in recent years. In fact, during 2010–2021, research in the building field was mainly driven by PCM application, as very different effects can be exploited depending on the relative placement of PCMs in a building or in specific climatic areas. Finally, research concerning the coupling of PCMs to ground-coupled heat pump (GCHP) technology is discussed. In fact, this technology, by employing the soil as a form of underground thermal energy storage, is one of the most promising and innovative LHTES architectures, as it is not influenced by seasonal effects.

## 2. PCMs’ Evolution in the Building Sector

### 2.1. The Early Stages: 2000–2010

At the very beginning of this period, the topics covered by the most significant research concerned PCMs’ basic properties, thereby almost neglecting the implications of outer factors for their efficient use as building material. In particular, these topics were heat transfer modeling [26,27], the conservation of temperature-sensitive materials, and thermal storage enhancement [14,28,29], as well as their first possible building applications, such a through impregnated wallboards [9,30]. Concerning basic properties, the materials were classified as PCMs considering mainly their significant liquid–solid phase change latent heat (at least 100 kJ/kg), and this led to a list over 150 organic and inorganic materials, both commercial and non-commercial. At this stage, the highest latent heats recorded were: 816 kJ/kg, attributed to a LiF–CaF_2_ (80.5:19.5) eutectic mixture among inorganic compounds; and 250 kJ/kg, related to paraffin wax, among organics. It is also worthy of note that Rubitherm (Berlin, Germany, www.rubitherm.de, accessed date 25 January 2022) and EPS Ltd. (Sonning, UK, epsltd.co.uk, accessed date 25 January 2022) were evaluated, during 2010, as the most relevant commercial PCM manufacturers in the world, with the highest number of available products (29 and 61, respectively). Nevertheless, several critical points common to the majority of PCMs arise, such as long-term stability, low thermal conductivity, density variation over the transition phase, corrosion, and subcooling or phase segregation. These drawbacks are particularly relevant for inorganic PCMs, including eutectic mixtures, such as LiF–CaF_2_, and limit their employment despite the higher value of their latent heat. In particular, LiF–CaF_2_ eutectic mixtures have shown a linear coefficient of expansion near to 30 × 10^−6^ K^−1^ and relevant changes over time (after 9 months) in density (−2%) and porosity (+2.1%) [31]. In addition, the numerical simulation and modeling of the heat transfer during the phase change, called the moving boundary problem, was also considered as a critical point to study, over several work cycles, to validate numerical simulation, due to a lack of experimental studies [14,26,32]. 

**Figure 2:**
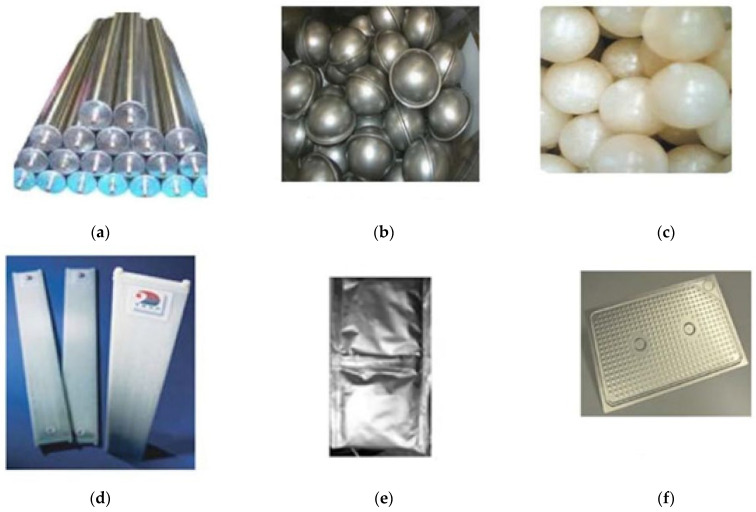
Examples of different container shapes used for PCM encapsulation: (**a**) tubes, (**b**) metal spheres, (**c**) PCM spheres, (**d**) rectangular PVC panels, (**e**) aluminum pouches, (**f**) flat panel [33].

As a possible strategy to overcome PCMs’ intrinsic low thermal conductivity, highly tailored encapsulation methods were investigated, as a promising way to increase PCMs’ heat transfer while avoiding large phase separations [28]. In particular, macro-encapsulation was analyzed as a viable and economical way of making PCM employment more efficient, giving them a self-supporting structure designed for their intended application. Examples of macro-encapsulation strategies are shown in Figure 2. They include metal tubes (Figure 2a), metal spheres (Figure 2b) that could be arranged to form a packed bed, PCM spheres (Figure 2c), aluminum pouches (Figure 2e), and rectangular flat panels in polyvinyl chloride (PVC) or aluminum (Figure 2d,f).

**Figure 3:**
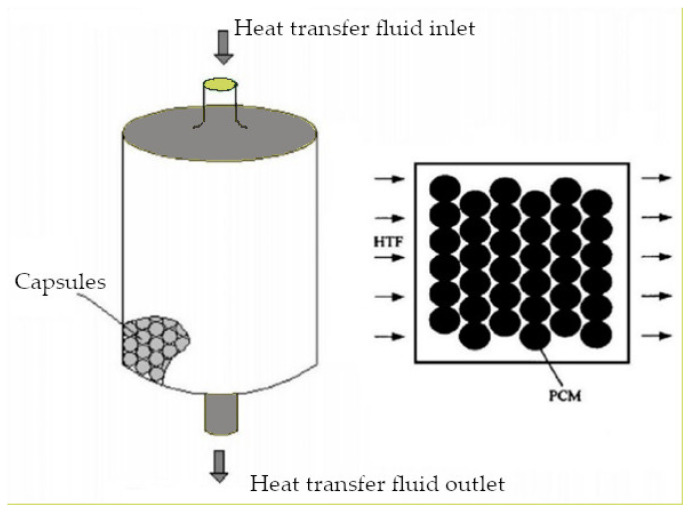
Packed-bed LHTES system diagrams with spherical macro-capsules [28].

These different tested geometries show different peculiarities. With flat panels and pouches, it is possible to achieve more surface area per unit of storage volume, having less weight and volume with respect to bulk PCMs. Subsequently, the solidification time results reduced to 4 h and the melting time reduced to 6 h, making it acceptable for free cooling by employing a low PCM thickness [34,35]. Nevertheless, the panel geometry was found to be superior to pouches as, under the same environmental conditions and by employing a commercial organic PCM, the first showed higher cooling power (+12.5%) and shorter melting times (−45%) [36]. Cylindrical PCM pipes are easier to fabricate, having the same heat transfer characteristics with respect to flat panels and a lower heat loss rate [33,37,38]. In fact, in this geometry, heat transfer takes place in the axial and radial directions, with an increased area of convective heat transfer. Concerning spherical geometry, PCMs or metals balls have larger surface areas per unit of volume compared to cylindrical geometry and heat transfer can be tailored by selecting the ball’s diameter. For example, balls up to 3 mm in diameter were tested, demonstrating that the solidification time is about 1.5 h, which is suitable for free-cooling applications [36]. In particular, a LHTES configuration based on PCM spherical geometry encapsulation (Figure 3) has been investigated as a possible solution to increase the heat transfer rate between the PCM and the heat transfer fluid (HTF) while avoiding direct contact between them [28]. However, the main problem of the packed-bed configuration concerns the complex modeling of both the heat transfer process between PCM and HTF considering the container and the PCM phase change inside the container itself [28].

When sphere geometry concerns restrained diameters (<5 μm), the micro-encapsulation definition is employed. Micro-encapsulation architecture, thanks to the larger heat transfer area between micro-encapsulated particles, ensures an even greater thermal conductivity with respect to sphere geometry encapsulation (or macro-encapsulation). However, a further encapsulation was needed, in these years, to collect all the micro-spheres, consequently reducing the micro-encapsulation’s beneficial effects on the final thermal conductivity dramatically [28]. In fact, further collection of the micro-capsules forces the majority of the heat transfer to occur through conduction, significantly limiting the overall heat transfer rate. In this context, for macro-encapsulated PCMs, the PCM-HTF heat transfer process modeling, as well uncertain long-term stability, requires more investigation due to micro-encapsulation architecture [39]. In addition, cost must also be considered, and at the early stage of the research this was among the most expensive storage methods for PCMs [28].

**Figure 4:**
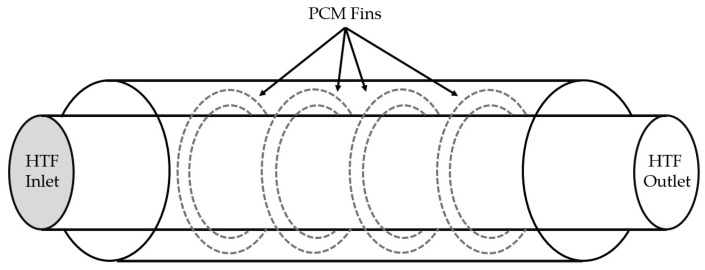
Ring-shaped fins added to a shell-and-tube LHTES unit [29].

To overcome micro-encapsulation issues, more complex geometries, such as the use of fins or extended surfaces in the PCM, to provide additional heat transfer surface, were studied in order to enhance thermal conductivity [29]. Nevertheless, it has been demonstrated that their employment must be finely tuned with respect to their final application, as the beneficial effect of fins decreases with increases in the heat transfer coefficient. For this reason, in most cases, fins must be included at the side of the PCM container and their use must be regulated if melting or solidification phases are to be enhanced [29]. In addition, fins can vary widely in material, number, and thickness, as these parameters are crucial to promote both conduction and convection processes during PCM phase change. From several studies, it was demonstrated that heat transfer during PCM melting is mainly ruled by natural convection, therefore, in this case, fins could be helpful to further promote the natural convection process [29]. In fact, generally, the same material as the PCM’s envelope is employed. Rectangular fins are the most frequently used due to their very simple geometry and low-cost manufacturing, but ring-shaped fins were also employed in configurations in which the PCM is placed around HTF tubes, as shown in Figure 4. Numerical models that compared the latter solution with a system without fins, demonstrated that the great majority of heat was conducted through the fins along the radial direction. For different ranges of mass flow rate and inlet temperature, a considerable amount of increase in the energy stored was observed due to the presence of fins, increasing with the fins’ number, up to +63% with 19 fins [29,40].

The impregnation of porous structures with PCMs was investigated as another viable way to increase their thermal conductivity. However, the final result depends not only on the materials’ thermal conductivity itself, but also on their mean pore size [29]. In fact, several numerical studies demonstrated a substantial decrease in the response time of the porous metal matrix-PCM system; consequently, a high porosity (up to 85%), for the obtainment of the highest possible performance, must be promoted. However, this solution raise concerns related to the final disposal of the porous structure, as the PCMs’ presence inside the porosity must be considered and evaluated at the end-of-life when recycling or landfill disposal are needed. The most commonly investigated materials were metals, e.g., aluminum or copper, and carbon-based materials, such as graphite, which presents a natural porous structure, or expanded graphite (EG) [29,41].

Metals can also be added to the PCM in the form of high-conductivity particles, or as metal structures (e.g., metal rings or screens). However, particle mass fraction must be carefully designed to enhance thermal conductivity, without lowering the thermal storage capacity of the PCM. In fact, metal structures usually occupy much more volume (20%) in the PCM with respect to other technologies, such as fins (7%). Copper, aluminum, and silver are the most studied metals for applications with particles, due to their high cost and enhanced thermal conductivity, while steel is preferred for metal structures or extended geometries. With metals, only paraffin-based PCMs can be used, because of the corrosion phenomenon due to salt hydrates [26,29]. Among carbon-based materials, characterized by high thermal conductivity and low density, carbon fibers have been considered, as they can be added in the PCM in form of fibers, brushes, or cloths. This material allows a good enhancement of thermal conductivity with a volume fraction of approximately around 1% and a reduction in solidification time of around 23%, but also presents high costs due to the need for a very uniform distribution of fibers in the PCM [29]. 

In these years, to overcome these challenges in PCM design and optimization, the mathematical and numerical modeling of phase change and heat transfer phenomena that take place in LHTES systems were approached first. Two main models and related categories were developed during the 2000–2010 period: those based on the first law of thermodynamics, which aimed to quantify the amount of stored energy; and those based on the second law, which aimed to quantify the usefulness of stored energy. Consequently, the two models were concern with energy and exergy analysis, respectively. Most of the studies in this period focus only on energy analysis, while exergy-based evaluation is less frequently considered, despite its value and complementarity with the first. This trend is due to the fact that most energy-based models are validated through experimental set-ups, while the acceptability of exergy-based models suffers from a lack of evidence against experimental data [27,41]. Historically, simplified analytical approaches were proposed with the so-called Stefan problem and Neumann method, as clearly reported by GSH Lock [42]. However, to comprehend the behavior of these phenomena, numerical codes are needed, as they formally implement and solve energy and heat transfer equations, based on different finite or volume element numerical approximations. 

The research on PCM applications in the building sector was at its very early stage during the period of 2000–2010. Trombe wall and building block applications were investigated with both organic PCMs, such as paraffin wax and stearic acid, and inorganic salt hydrates. In this context, several studies demonstrated that for a given amount of heat storage, the addition of PCM (~20% of the weight being paraffin) requires less Trombe wall volume (−20%) and weight. At the same time, concrete and wallboard functionalization (direct mixture), mainly with mainly organic PCMs, such as paraffin waxes or fatty acids, were considered [6,30]. Despite the promising results of these studies in terms of good heat transfers, some critical issues must be highlighted. The most important of all was the small number of suitable commercial materials, which must have both a transition temperature falling in the human comfort range (20–32 °C) and a reasonable cost of employment in amounts high enough to be mixed with concrete [6,9]. In addition, to enhance the free cooling phenomenon, the combination of at least two PCMs was suggested to allow year-round thermal management [33]. In fact, free cooling takes advantage of low night temperatures to store coldness in building materials enriched with PCMs, or in LHTES units, and release it for indoor cooling during the daytime and, for this reason, is strongly influenced by seasonal variation. Thereafter, it is almost impossible to choose only one PCM as it would melt/solidify only for a part of the year.

### 2.2. Further Material Evolution from 2011 to 2015

In this period, in contrast with the previous decade, discussed in Section 2.1, more efforts were dedicated to the specific application of PCM technology in the building sector, by further considering the main issue related to a specific technology (e.g., micro-encapsulation) or applications in different building elements (e.g., walls, mortars, glazing units). According to Cabeza et al. [43], and as shown in Table 2, at the beginning of this period (2010), many substances were investigated as PCMs for specific applications and temperature ranges in the building sector. Subsequently, in this period, PCM classification also started to take into account applications in the building sector. As shown in Table 2, most of these substances are constituted by organic materials, mainly paraffins and fatty acids, due to their high performance/cost ratio compared with the other PCM categories.

**Figure 5:**
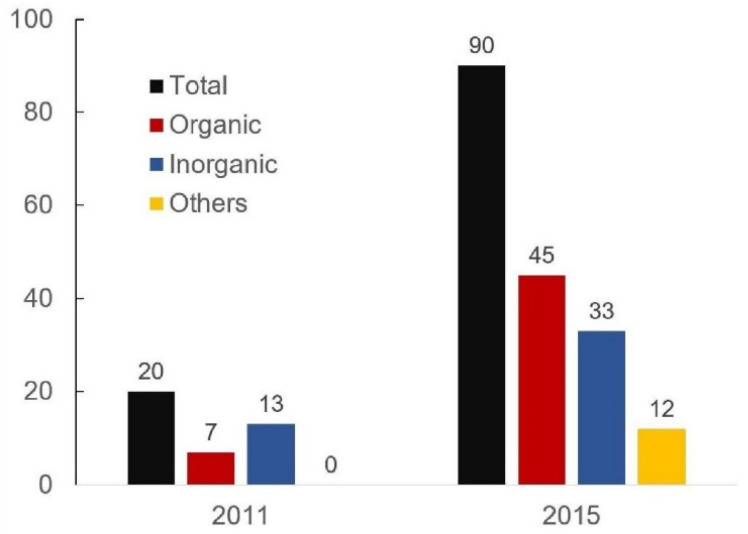
Comparison between 2011 and 2015 of commercial PCMs useful for building applications, in the 15–33 °C range [43,45].

As shown in Table 3, the number of commercial PCMs available in 2010 was limited to 88 products, which is quite low if compared with the 244 total PCMs in Table 2, as confirmed by Zhou et al. [44]. In addition, it can be noted that at this stage, no commercial PCM had been developed for hot-water applications; nevertheless, the great majority of the total number of PCMs were devoted to this application (Table 2). A possible reason for this trend is that commercial products had been developed for more recent and advanced applications at that time, while hot-water applications were the most consolidated. Later studies reported a strong acceleration of the development of new PCMs dedicated to the building sector. A comparison between commercial products is represented in Figure 5, which shows that in the same temperature range (15–33 °C), the number of commercial PCMs grew from 20 to 90 in 4 years, from 2011 to 2015. The growth concerns all PCM categories, but organic substances show the highest increase during these years with great variability in the technology used, e.g., tubes or mats filled with PCMs, gypsum boards, floor tiles, windows, heat exchangers, or ventilation systems with PCMs [43,45].

Some of the first studies in this period (2011–2012) still focused more on the issues concerning PCMs’ intrinsic properties than on the problems facing their application in building materials [43,44]. The main critical points that arose in this period were: the need to further enhance the heat transfer due to PCMs’ low thermal conductivity (from 0.2 W/m⋅K for paraffins to 0.5 W/m⋅K for hydrated salts); the long-term stability of PCMs with an increasing number of thermal cycles; the compatibility between the PCM and its container, such as corrosion between salt hydrates and metal and the dimensional stability of plastics containing organic PCMs; and the phase segregation or subcooling of salt hydrates [43,44]. In fact, in this period, investigations tried to exploit salt hydrates’ peculiarities due to their intrinsic higher phase change enthalpy, by reducing their possible drawbacks. Later studies in this period show that these problems were not completely solved for specific categories of PCM, even in the following years. 

In fact, the long-term stability of paraffins was assessed, as all the materials tested showed almost no changes in their properties after a number of thermal cycles, between 300 and 5000 [46]. Different studies demonstrated that the stability of fatty acids depends instead on their purity degree, as compounds with a purity degree between 90% and 95% (e.g., stearic acid, palmitic acid, myristic acid, or lauric acid), showed a latent heat drop after 900–1200 thermal cycles [46]. Phase separation and the supercooling of salt hydrates were improved, respectively, by employing thickening agents (e.g., attapulgite clay, polyvinyl alcohol, silica gel) and nucleating agents (e.g., Borax, sodium pyrophosphate decahydrate), subsequently leading to improved long-term stability (e.g., CaCl_2_ 6H_2_O can reach 1000 working cycles without phase separation by addition of NaCl and H_2_O) but with a more complex PCM formulation to keep under control during their fabrication [45,46].

With respect to the previous period (2000–2010), in these years (2011–2015), more specific ways were found to incorporate PCMs into building elements. According to Zhou et al. [44], five main methods can be defined, as follows:

- Direct incorporation: the PCM and the building material are physically mixed at the solid state.
- Immersion: a building element, e.g., bricks or concrete, is dipped into a liquid PCM, which enters into the open pores thanks to capillary forces.
- Macro-encapsulation: the PCM is encapsulated inside a container with a variable shape.
- Micro-encapsulation: the PCM is encapsulated through a thin film of a polymeric material to form particles with low dimensions, with a diameter lower than 5 µm.
- Shape stabilization: the formation of a composite material (SSPCM, shape-stabilized phase change material), where the PCM is the matrix, while another material acts as the reinforcement (e.g., HDPE).

Among the listed methods, micro-encapsulation was particularly investigated, as new improvements in this field are related to innovative and more specific manufacturing technologies. In fact, compared with macro-encapsulation, the safety of PCM handling, due to the reduced dimension, must be carefully taken into account; the development of new, environmentally safe materials became a significant trend during this period, as well as the improvement of the stability of the microencapsulated PCM by up to 100 cycles [44]. In addition, since the overall thermal conductivity can also be potentially lowered by the material used for the shell due to the restrained PCM mass fraction, thinner encapsulations were investigated in these years through the employment of high-molecular-weight polymeric film [28]. For this reason, the main categories of PCM considered for micro-encapsulation are paraffins (e.g., heptadecane or octadecane) and fatty acids (e.g., eicosanoic or stearic acids) that have intrinsically higher thermal conductivity with respect to salt hydrates [47,48]. 

Taking into account the specific application of PCMs in the building sector, although the sector involves different building elements, wallboards, concrete, floor and ceiling systems, windows, and shading elements were investigated, as these construction elements offer large areas for heat transfer within building enclosures. Nevertheless, between 2011 and 2015, the number of empirical studies on these applications was quite limited, particularly for windows and shading units as they are a weak link between internal and external conditions in buildings, and they are quite challenging to simulate or replicate in laboratory conditions [44,49,50]. A greater number of studies also concerned the numerical simulation of the inclusion of PCMs in building elements, particularly walls. These studies mainly aimed to evaluate the optimal phase change temperature needed, or the energy consumption and thermal performance related to the PCM inclusion in the building, depending on seasonal variation and geographic location [44]. The results demonstrate that, at this stage of the research, numerical approaches driven by specific index estimation, such as thermal resistance, heat storage coefficient, and index of thermal inertia, are able to estimate temperature variation with good approximation (~2 °C) [44]. 

Wallboards and gypsum plasterboards functionalized with PCMs have been investigated as cheap lightweight materials capable of enhancing the thermal comfort and management of buildings through the reduction of internal temperature fluctuations [44,51]. Many studies consider only organic PCMs with a phase change temperature between 18 °C and 30 °C, such as PEG 600, butyl stearate, micro-encapsulate paraffin, or capric acid and lauric acid mixtures. The matrix or supporting material of the panel is usually made of gypsum, polycarbonate, or high-density polyethylene (HDPE). Empirical studies demonstrated that during winter, these wallboards are capable of reducing the indoor wall temperature fluctuation by 1.15 °C and reducing the heat dispersion towards the outside environment [51]. Wallboards used for cooling purposes allow a decrease in the indoor temperature during the summer peak of up to 4 °C, but this is possible only if the PCM’s solidification (up to 60 wt% of micro-encapsulated paraffin) is reached during the night; consequently, at sufficiently low temperatures, this application is strongly dependent on seasonal variation and temperature fluctuation [44,48,50]. In addition, it has been demonstrated that PCMs’ integration in wallboards is strongly affected by the efficiency of the manufacturing technique, and on the PCM/matrix ratio, as very different particle size distributions can be achieved, leading to different heat transfer efficiencies. In fact, a very restrained diameter of 0.16 μm can be achieved through the chemical route, such as emulsion polymerization, whereas greater diameters, up to a few millimeters, can be achieved with physical methods, such as spray drying [48]. The choice of the shell material also plays a very important role, driving microencapsulated PCMs’ morphologies, shell mechanical strength, heat capacities and thermal stabilities. Among the organic materials that are easiest to manipulate for microencapsulation fabrication, the most frequently employed are: polystyrene, polyurea, polymethyl methacrylate, arabic gum, amino resin, urea formaldehyde resin, melamine, and formaldehyde resin. Nevertheless, their employment should be restricted due to their flammability, toxicity, and low heat conductivity. By contrast, the inorganic materials employed are mainly silica-based due to their desirable properties, such as their chemical and thermal stability, flame retardancy, high storage capacity and good compatibility with building materials [48]. Due to this complexity in the period investigated, a new category of PCMs arose, named composite PCMs. Moreover, direct solar gain and the ventilation rate should be considered together as environmental temperature fluctuation, as they can affect the effective temperature measured on the wallboard, leading to over or underestimation of the PCMs’ performance. Finally, it must be noted that, in several studies, the amount of the building element (e.g., a wall) covered with PCM, or with PCM added to it, is not always specified, and this leads to an increased difficulty in quantifying the real performance of the PCM, which also makes it more challenging to compare between different studies [10,44,48,49,51,52].

The integration of PCMs into floors and ceilings is usually intended to charge the materials with heat or coolness during the night and release them during the day, shifting the peak of electricity consumption to the off-peak period and allowing the use of cheaper energy. Wallboards have also been used in ceilings, coupled with active systems to enhance indoor thermal regulation. Metal containers (typically iron or steel) filled with salt hydrates or micro-encapsulated paraffin mixed with gypsum or rock wool were placed on the ceiling and coupled with active systems, as a ventilation system or a capillary system of water tubes. The results demonstrated that these systems are able to store coolness during the night and release it during the day, reducing the maximum peak temperature by up to 2 °C [48]. A similar principle is used in underfloor heating or cooling systems, which could be coupled with PCM plates to store heat or coolness [10,44,48].

**Figure 6:**
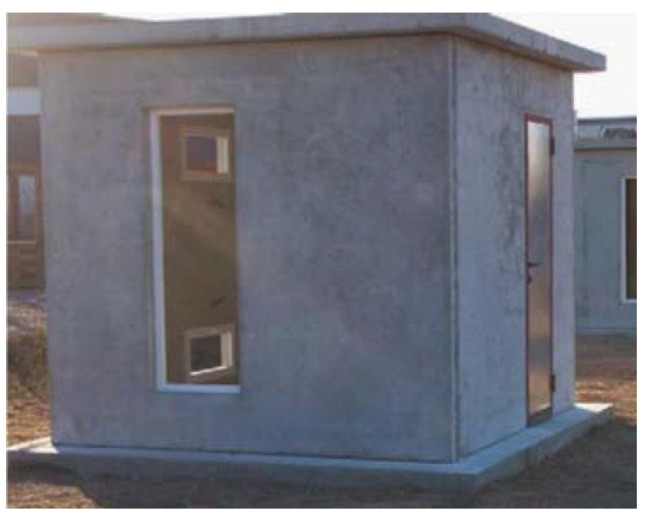
Test of concrete cubicles enhanced with PCM [51].

Different studies investigated the addition of PCMs into concrete matrices, as they are characterized by a high thermal mass, which allows the storage of thermal energy. Furthermore, for this application, usually, only organic PCMs are considered, e.g., paraffin, butyl stearate, dodecanol, and tetradecanol [53]. One of the main critical points investigated, in greater depth than in previous years, was how to promote the addition of PCMs into the concrete matrix, avoiding an excessively strong decrease in mechanical properties. In fact, although the addition of micro-encapsulated PCMs seems to consistently result in the worsening of mechanical properties, the final product is still appropriate for several building purposes. It has been demonstrated that a compressive strength of over 25 MPa and a tensile splitting strength of over 6 MPa, without any variation after 6 months, can be reached by adding a 5 wt% of commercial PCM called Micronal from BASF [44,53]. On the other hand, other studies report a 13% decrease in compressive strength for each 1% of micro-encapsulated paraffin added to the matrix, or a decrease equal to 32% for a 5% addition of micro-encapsulated PCM [48,53]. The difference among these results must be founded in the fact that, during these years, specifically tailored commercial materials have been developed in order to limit the concrete’s property loss. The loss of workability and the higher costs connected to the addition of PCM to concrete are additional problems, and the limited percentage of material that could be added could result in a low increase in the total heat storage capacity for concrete [49]. Furthermore, micro-encapsulated PCM addition between 1% and 5% grants an increase in concrete-specific heat capacity; it is also the cause of lower thermal conductivity. Experiments performed on concrete cubicles (Figure 6) with 5% paraffin-based commercial PCM demonstrated that summer peak temperatures could be lowered by up to 3 °C with PCM addition when compared with concrete only [51,53]. On the other hand, it is not clear whether this lower peak was due to the higher thermal capacity or to the lower thermal conductivity of the material, although this performance can be repeated every day only if the PCM is allowed to completely solidify during the night [44,48,49,51,53]. In this context, PCM leakage out of micro-capsules must also be considered as a potentially critical issue, not only for overall technical performance but also for environmental safety [51].

In these years, as previously suggested, a limited number of studies also investigated more sophisticated ways of introducing PCMs into building elements, such as windows or shutters. Window improvement could be a viable way of reducing heat loss during winter, while shutters integrated with PCMs could reduce the amount of heat that enters buildings during summer, as they are the main paths in charge of heating loss or absorption, through solar radiation, of the overall building structure. For these reasons, some studies focus on the addition of PCMs to glazing and shading elements. The use of salt hydrates (with a melting interval of 24–29 °C) with conventional glass for windows is reported, as they are both inorganic materials and are, therefore, more chemically compatible [45,49]. The two materials showed good compatibility and a reduction in heat losses equal to 30% compared with conventional glass, even though leakage problems are reported [49]. Other studies reported that the use of salt hydrates in windows during summer could reduce the gained energy amount by up to 50% [45,49]. Nevertheless, one general issue is the transmittance of salt hydrates with respect to visible wavelength, a property that greatly depends on the physical state of salt hydrates. In fact, even in the most optimized systems, salt hydrate-enhanced windows cannot guarantee a clear view of the environment beyond them. The use of salt hydrates (with a melting interval of 26–30 °C) is also reported for vertical slats employed as shading units. Their use in summer granted lower indoor temperatures (between 1 °C and 2 °C), even though the salt hydrate phase change during the night was prevented by the outside temperature, without the use of a mechanical ventilation system [45,48,49].

During the same years (2011–2015) there was a growing but limited interest related to the environmental sustainability of PCMs to determine the global benefits of such solutions, as also demonstrated by an increasing interest in more advanced microencapsulation techniques and materials. Consequently, the contribution of the relevant life cycle phases was included in some studies, and the possible burdens of shifting from one life cycle phase to another was taken into account due to the use of these emerging technologies within the construction systems. A life cycle assessment study of an alveolar brick construction system, incorporating salt hydrates SP-25 A8 encapsulated in CSM (compact storage module) panels, demonstrated that a reduction in the environmental impact was produced due to the energy savings achieved during the operational phase, compensating for the increase in the environmental impact due to the use of PCM [54]. Furthermore, the environmental impact due to the inclusion of ester as PCM in building envelopes was studied and compared to other PCMs (paraffin and salt hydrates). The results demonstrated that the impact of ester used as PCM slightly improves the effect obtained in the case of using salt hydrates during the manufacturing impact. On the other hand, the use of salt hydrates or ester as PCM led to impact reductions of 9% and 10.5% respectively, compared to the case of using paraffin [55].

### 2.3. The More Recent Evolution from 2016 to 2021

In these years, studies were mainly focused on the different applications of PCMs in the building sector by exploiting their peculiarities in specific technologies, as macro- and micro-encapsulation, or on specific building elements, as mortars.

According to Table 4, which reports a summary of the most studied materials according to Akeiber et al. [19], Liu et al. [56], Singh Rathore et al. [57], and Da Cunha and De Aguiar [58], the majority of experimental studies still consider organic PCMs. Paraffins (e.g., hexadecane, octadecane, and eicosane) are the most investigated, followed by fatty acids (e.g., capric acid, lauric acid, and palmitic acid). The phase change temperature of PCMs usually varies between 18 °C and 30 °C, but there are some applications with higher temperatures, between 37 °C and 43 °C, according to climate changes and particular needs. The same trend was also confirmed by Song et al. [59], who list the applications of PCMs in different parts of the building envelope, namely walls, ceilings, and floors. However, the distribution of studies between different building elements is not equal. According to Zhu et al. [60] and Da Cunha and De Aguiar [58], the majority of studies concern wall applications (approximately 60% of studies), followed by ceiling and floor applications, which mainly include the introduction of PCMs into concrete, gypsum boards or panels. Considering Table 4, macro-encapsulation techniques were still favorable with respect to microencapsulation, due to the still-high cost of the latter.

An additional focus on the experimental studies performed on PCM applications between 2019 and the first half of 2021 is presented in Table 5 (passive applications) and Table 6 (active applications). The trend presented by the studies in Table 4 is mainly confirmed, as paraffins were the most studied PCMs during these years, with some exceptions, such as tetradecanol and hexadecanol or coconut oil. All the materials studied are organic, with a small number of exceptions that consider two salt hydrates, namely CaCl_2_ ∗ 6H_2_O and MgCl ∗ 6H_2_O. Nearly half of the studies still consider macro-encapsulated PCMs, while micro- or nano-encapsulated PCMs are a minority, as shape-stabilized PCMs. The phase change temperatures range from 17 °C to 44 °C, and walls are again the most studied building elements; however, the inclusion of PCMs in the ceiling and floor is also studied. Most of these studies investigate the performance of building elements in passive conditions in field tests, attempting to understand whether the inclusion of PCMs alone could prevent or minimize the use of active systems to regulate indoor thermal conditions in different climates, as well as avoiding energy consumption. 

**Figure 7:**
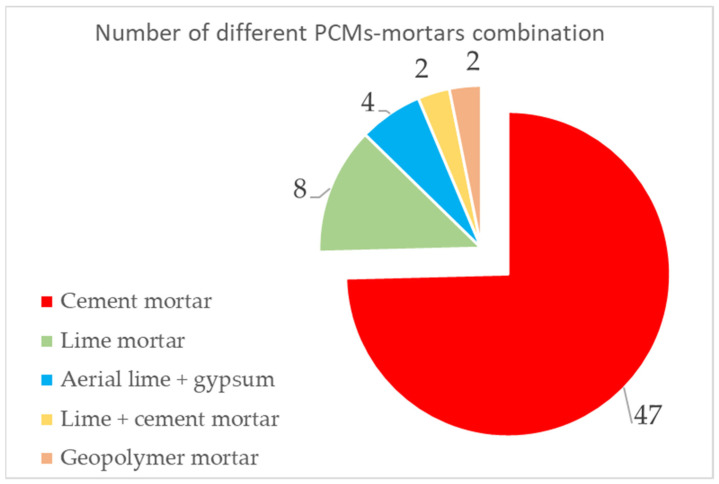
Number of different combinations of PCMs and mortars investigated in the period 2016–2021 [79].

As previously suggested, in this period, one of the principal innovations was due to the introduction of PCMs to mortars. The introduction of PCMs into concrete and mortars was reviewed by Rao et al. [79] and Berardi and Gallardo [80]. Different kinds of mortar were considered, but cement mortar was the most studied, as shown in Figure 7, compared to other types, such as lime mortar, aerial lime, and gypsum, or geopolymer mortar.

From the analysis of these studies, paraffins were the most frequently studied of the PCM categories with the potential to be incorporated into mortars, as they do not disturb the hydration reactions. On the other hand, common direct incorporation methods, including the wet mixing or immersion technique, usually imply a high risk of leakage of the PCM in its liquid phase, or an interaction with the matrix during its lifetime, and this problem is quite critical for organic PCM employment [58,80]. Other direct incorporation methods tested require a supporting material, which is often cheaper than those used for macro or micro-encapsulation, to avoid PCM leakage [79,80]. Silica fume and expanded graphite were studied as supporting materials, as they also enhance the thermal conductivity of the PCMs. Other materials studied were expanded perlite or vermiculite, lightweight aggregates (LWA), or rice husk aggregates, as they are chemically compatible with mortars [79,80]. The use of paraffin and expanded graphite showed good chemical compatibility with lime mortar, but involved too high a cost due to the micro-encapsulation process [79]. LWAs (based on clay, pumice, diatomite, and expanded vermiculite or perlite) were enriched with PCMs through the vacuum impregnation technique, which enables the absorption of PCM up to 73 wt%, and then coated with epoxy or polyester resin, or limestone powder, to avoid leakage [80]. However, it was demonstrated that the use of these coatings reduced the thermal conductivity of the whole material, while the addition of LWAs enriched with PCMs is reported to reduce the compressive strength of mortars from 10% to 72%, depending on the LWA material and the PCM percentage added [80]. Nevertheless, the use of expanded perlite (30 wt%) was successfully tested to control the leakage of paraffin [80].

The use of micro- and macro-encapsulation was also studied to add PCMs into mortars. For the first, commercial polymeric microencapsulated PCMs were employed, often as a replacement of a part of fine aggregates used in concrete or mortars, such as sand. The results showed that the amount of micro-encapsulated PCMs should not exceed 6 wt% of concrete (which corresponds to 10–12% of volume) to limit the reduction in the workability and compressive strength of the whole material, which is usually around 40% for the compressive strength when the PCM weight percentage is higher than 3% [80]. The use of micro-encapsulated PCMs also reduces the thermal conductivity of the material with respect to the concrete without any PCM. In fact, despite the higher specific surface of micro-capsules with respect to macro-capsules, they have lower thermal conductivity than common sand. Moreover, their addition increases porosities of the concretes, which are filled with low-conductive air. This effect causes a drop from 25% to 50% in the thermal conductivity of concrete when a 5% mass of PCM is added [58,80]. Macro-encapsulated PCMs employ stainless steel to encapsulate the PCM, leading to many advantages, such as lower leakage compared to micro-encapsulated PCMs, and the unmodified compressive strength of the mortar [79]. The use of other materials, such as PVC or other polymers, should allow the use of inorganic PCMs not compatible with metals, such as salt hydrates. However, there are almost no studies on them, probably due to several critical issues such as phase separation, supercooling, and the lack of long-term stability [58,59,79,80].

Table 7 lists other experimental studies focused on the inclusion of PCMs in concrete and mortars between 2019 and 2021. With respect to the studies presented in Table 5 and Table 6, no salt hydrates are considered, but a greater variety among organic PCMs can be appreciated. In fact, a consistent number of them belongs to alcohols or fatty acids, as well as paraffins. The majority of PCMs were included in mortars in a shape-stabilized form, mainly obtained through the impregnation of natural porous materials (e.g., perlite, pumice, diatomite, vermiculite). Other studies evaluated the inclusion of PCMs in microencapsulated form, and only a few works investigated the direct inclusion in the mortar [81,82,83]. Portland cement is the most studied binder, but alkali-activated cements and geopolymers also seem to be considered as viable alternatives. No problems due to chemical or physical interactions between PCM and mortar/concrete emerged; nevertheless, the percentage of PCM must be limited (up to 20 wt% in the mortars), mainly to control the decrease in mechanical properties, such as compressive strength and flexural strength [82,84,85,86,87]. Instead, the decrease in the workability of fresh mortar/concrete is usually solved by adding more water in the initial mixing of the materials or by using a superplasticizer [81,84,88,89].

In this period (2016–2021), particular attention was put on applications related to specific seasonal temperatures and variations, leading to a very peculiar situation that required the use of multiple PCMs (with different phase change temperatures), or well-tailored PCMs, as previously detailed for microencapsulated PCMs. In addition, following both the climate change issues all over the world and the acceleration of the building sector in countries (e.g., India and Saudi Arabia) with extremely hot climates (up to 45 °C), PCM investigation specifically tailored for particular climates is a practical and actual need [8,19,57,99,100].

In this context, a direct contribution is also given to the environmental aspects, through a more focused investigation concerning the life cycle assessment analysis performed on PCM applications in constructions. Nevertheless, the environmental impact of PCMs’ use in the building sector is still an aspect that needs to be studied and that could have important outcomes on the choice of materials and technologies [101]. For instance, one of the advantages of macro-encapsulation over micro-encapsulation is the easier separation between the PCM and the shell material, which simplifies their recovery at the end of life [56]. This perspective was investigated by Kylili and Fokaides [102], which studied the impact on the environment through a literature review on the life cycle assessment (LCA) studies performed on PCM applications in buildings. For the most part, these studies used the EI99 Environmental Impact Assessment methodology and focused only on some of the life cycle stages of the investigated materials, particularly the manufacturing, operational, and disposal stages. Compared to organics (such as paraffins, fatty acids, polyethylene glycol), inorganic PCMs (such as salts hydrates) are less dangerous as they are chemically stable and can be recycled (Table 8). Other variables were the seasonal conditions, as some studies considered only the summer or the winter season, and the systems’ lifetime, which varied between 50 and 100 years. The results demonstrated that PCMs incorporated in building materials have a lower impact on the environment compared to reference cases, without any addition of PCMs. However, these LCA findings are highly dependent on the scopes and considered phases. The majority of studies did not consider the lifetime duration of PCM-enriched building materials. Between the manufacture, operational, and disposal stages of these materials, usually only one or two are analyzed. The results showed that the use of PCMs in building materials is not always environmentally friendly and is highly dependent on life stages. For instance, one of the studies highlighted that the addition of PCMs in a ventilated double-skin façade is environmentally efficient only if the operational period of the façade lasts at least 31 years. Below this value, the environmental impacts due to manufacturing and the disposal of the façade would overcome the benefits provided by the addition of PCM [102].

Another way to reduce the environmental impact of PCMs is to consider biodegradable substances or materials derived from wastes. Some studies, such as those reported in Table 9, consider natural substances as PCMs, e.g., coconut oil or beeswax [103,104]. Other studies, such as those listed in Table 9, try to use natural materials (e.g., wood) or wastes (e.g., fly ashes, glass scraps) as supporting materials to produce SSPCMs.

In addition, to make PCM-enhanced building materials more attractive for the market, in this last period the need arose to perform comprehensive cost analyses and economic evaluations on the applications of these materials in the building sector, as highlighted by different authors [8,19,57,79]. Furthermore, to achieve this scope, the adoption of common and standardized procedures should be considered. Firstly, they should concern all the procedures used to design and produce PCMs-enriched materials for the building sector, such as mortars. Secondly, the methods used to measure the thermal properties of PCM-based materials should also be standardized. For example, one of the most common methods currently used to evaluate the melting temperature and phase change latent heat is differential scanning calorimetry (DSC), which analyses samples with a mass in the order of a few milligrams. This amount is not enough to give a good measure of heterogeneous materials, such as a mixture of cement mortar and micro-encapsulated PCM leading to a mismatch between measured and real values of the investigated thermal property [57,79,80]. To enhance the measurements’ standardization, other methods could be used, such as the T-history method or the use of a heat flow meter apparatus, which is suggested both by the Quality Assurance RAL-GZ 896 [116] and the Active Standard ASTM C1784 [117] documents.

## 3. Future Challenges: PCM–GHE Coupling

Among renewable energy technologies, air-source heat pumps (ASHPs) and ground-coupled heat pumps (GCHPs) have been regarded, in recent years, as reliable and efficient solutions for residential air conditioning applications; their use has also been driven by their use of renewable-source electricity, which avoids the use of primary energy [118]. GCHP technology uses the soil as underground thermal energy storage and represents a viable alternative to common ASHPs. In fact, GCHPs do not suffer from seasonal effects, which lower both the efficiency and heating/cooling capacity of ASHPs. In fact, ASHPs have a low initial installation cost and are easily applicable; however, they can suffer due to low ambient temperature in winter, frosting on the outdoor evaporator, and hot ambient temperatures in summer. When ASHPs encounter these extreme conditions, their overall efficiency and heating/cooling capacity is reduced, and defrosting can waste more than 12% of the full seasonal heating load [119]. By contrast, GCHPs can achieve higher efficiency than ASHPs by employing underground thermal energy storage (UTES) to provide more moderate operating temperatures for the heat pump. GCHPs can be installed at any location where drilling or earth trenching is feasible. Besides their higher efficiency, their cost-effectiveness is mainly penalized by their ground heat exchangers (GHEs), since the very low soil thermal diffusivity requires larger sizes, which leads to higher initial costs [120].

Ground heat exchangers usually consist of a buried piping loop, which can be installed in vertical boreholes or shallow horizontal trenches (also referred to as VGHEs and HGHEs, respectively). The use of VGHEs can benefit from the relatively stable temperature in the deep ground, beyond 20 m. However, a substantial thermal imbalance between seasonal heating and cooling loads can result in a larger temperature rise or fall in the ground over the year. The common solution to mitigate this problem is the significant oversizing of the VGHE, which ultimately reduces the economic feasibility of the project. Otherwise, a supplemental thermal source (air or solar) may be integrated into the ground loop to smooth the imbalance as well [121]. By contrast, an HGHE is generally installed in shallow ground up to 2 m deep. Therefore, it is more subject to variation in the environmental conditions; however, this can actually mitigate the above-mentioned issue of thermal imbalance in mild climate [122]. A prominent advantage is that the installation of HGHEs only needs a fairly simple trench excavation, and the removed soil can be directly re-employed for backfilling. Additionally, installation at shallow depths allows considerable freedom in the design of the GHE geometry, and the geologic characterization is not as essential as it is for HGHEs [123]. Further, HGHEs may allow the easy mixing of the backfilling material with PCMs for higher performance. In view of these distinctive advantages, the research interest in the development of advanced HGHEs for building applications has grown in recent years.

The GCHP technology is not new, as its first applications are reported in the first decade of the twentieth century; nevertheless, the problems exposed previously concerning installation and functioning strongly hindered its wide spreading. For this reason, the integration of PCMs in vertical and horizontal GHEs is still a recent field of research and there are few studies concerning this technology, most of which are numerical studies. Table 10 shows the different studies that analyzed PCMs’ integration in different parts of a GCHP. Between 2012 and 2015, there were only works dedicated to a numerical analysis of PCM integration in the GHE backfilling material, while from 2016 to now only a few studies performed empirical investigations. Several studies aimed to assess which were the advantages and issues related to PCMs’ addition in the backfilling material.

Dehdezi et al. [124] analyzed the thermal properties of a microencapsulated paraffin wax-soil mixture and simulated numerically the effects of the PCM addition on the soil temperature around a shallow-ground GCHP. The addition of microencapsulated paraffin wax caused a reduction in soil thermal conductivity and diffusivity, with a 3 °C lower temperature variation in the soil and a higher (17%) coefficient of performance (COP) of the GCHP. Similar findings were highlighted by Bottarelli et al. [125], who studied the coupling of a novel shallow GHE, a so-called flat-panel, with microencapsulated paraffin mixed directly with the backfill soil. The presence of microencapsulated paraffin smoothed the ground temperature variations due to seasonality and GCHP action, also ensuring a higher COP for the GCHP itself. To mitigate the problem concerning low thermal conductivity, Wang et al. [126] compared, through a numerical simulation, the separated use of soil, a mixture of n-decanoic acid and lauric acid (7:3 *v*:*v*) as pure PCM, and PCM with the addition of metal particles, as grout in a vertical GCHP. The results demonstrated that the replacement of common soil with the n-decanoic acid/lauric acid mixture could reduce the amount of land area needed to install the GCHP by 18%, while the use of enhanced PCM led to a reduction of land area of 29%. The importance of thermal conductivity enhancement for the PCM grout was also confirmed by Chen et al. [127], who investigated paraffin as a PCM. However, thermal conductivity also depends on soil characteristics, such as groundwater presence, which increases it. Qi et al. [128] investigated the influence of different PCMs (RT27, a commercial paraffin from Rubytherm and a mixture of capric acid and lauric acid (66:34)), with and without the addition of metal particles, on the backfilling grout of a vertical GCHP, finding that the thermal radius effect was smaller using PCMs independently of the metal particles’ addition. Zhang et al. [129] compared the performance of a common vertical GCHP with a shallow-ground GCHP surrounded by a cylindrical underground thermal battery (UTB) filled with water and a commercial mixture of salt hydrates as the PCM. The results demonstrated that the UTB guarantees more stable temperatures of heat transfer fluid with a ten times shorter borehole compared to a conventional vertical GCHP. Bonamente and Aquino [130] carried out a performance analysis, through numerical simulation, and a life cycle assessment study of a vertical GCHP coupled with an upstream LHTES for space conditioning. The analysis was based on a prototype and considered the impacts of the system from the production of input materials to the end-of-life of the GCHP. The LCA analysis highlighted the high relative impact of the LHTES but also the capacity to lower the whole plant energy consumption and environmental impact.

Since 2016, only a small number of studies have experimentally verified the effect of PCMs in the backfilling material of a GCHP. Li et al. [131] simulated the use of crushed stone and shape-stabilized PCM (a mixture of decanoic acid and lauric acid (3:2 *v*:*v*)) concrete enhanced with silica and graphite as grout for a U-tube vertical GHE. The model’s effectiveness was proven through an experimental measurement of the backfilling material at a laboratory scale. The simulation proved that PCMs increase the heat storage capacity of the borehole and lower its influence radius on the temperature of surrounding soil, while graphite and silica enhance the heat exchange. Yang et al. [132] performed an experimental thermal performance analysis of a borehole GHE on a laboratory scale to validate the numerical simulation of a vertical GCHP with PCMs (a mixture of decyl acid and lauric acid (66:34 *v*:*v*), and oleic acid) in the borehole backfill. The results confirmed that PCM employment enhances the GCHP performance and lowers soil temperature variations. Finally, Barbi et al. [133] studied experimentally the effect of PCMs’ addition in the backfilling material, common silica sand, of a shallow-ground GCHP. The results of tests on a laboratory scale demonstrated that the presence of sand promotes heat transfer. The sand showed a change in some physical properties, such as lower grain size, after some thermal cycles. However, the thermal performances of the mixtures remained constant through cycles.

Other studies investigated the integration of PCMs with GCHP elements other than the backfilling material. Zhu et al. [134] analyzed the energy and economic performances of a hydrate sodium sulfate as PCM cooling tank coupled with a vertical GCHP for the conditioning of an office building. This work demonstrated that the use of PCM storage is useful, as it guarantees lower energy consumption and operational costs for the GCHP. However, it also requires a careful design based on the building type, location, and system utilization criteria. Pu et al. [135] compared numerically the performances of pure water and water enriched with micro-encapsulated n-Octadecane as the PCM, and as the HTF in a horizontal three-shaped and straight tube GCHP. The results demonstrated that the use of micro-encapsulated PCM enhances water performance, but this depends on the GCHP’s design. Other few works studied the implementation of micro-encapsulated n-Octadecane slurries in the HTF of GCHPs to enhance its thermal properties. Kong et al. [136] performed a field test with micro-encapsulated methyl stearate. To avoid the risk of clogging in the GCHP system, the ground loop used only water as HTF, while the PCM circulated in a separate loop, placed between the GHEs and the heat pump. Despite its low latent heat, the PCM showed high durability and better performance as an HTF than pure water, but its direct use in the ground loop is yet to be verified due to clogging issues.

**Table 10:** Studies on the integration of PCMs in different parts of GCHPs. Num = numerical study, exp = experimental study, H = horizontal GHE, V = vertical GHE.

Reference	Study	Type	GHE	PCM	Melting Point (°C)	Latent Heat (kJ/kg)	PCM Employment
[124]	num	one-dimensional finite difference transient heat transport model	H	micro-encapsulated paraffin	26	160	backfilling soil
[126]	num	three-dimensional	V	decanoic acid and lauric acid mixture.	25	/	GHE’s borehole as grout.
[125]	num	two-dimensional	H	water, micro-encapsulated paraffin	0, 26	/	backfilling soil
[134]	num	modified composite model	V	hydrate sodium sulfate (type 47)	8.3	95.4	GCHP
[137]	num	computational fluid dynamics simulations	V	RT6 RT27	8.0–8.5 25.0–25.5	140 146	GCHP
[131]	num	three-dimensional unsteady model	V	decanoic acid and lauric acid mixture	20.15	128	backfill material in a GCHP
[128]	num	three-dimensional finite element model	V	paraffin RT27. decanoic acid and lauric acid mixture (66:34)	28–30 20.4	179 138.8	GHE’s borehole grout.
[136]	exp	/	V	micro-encapsulated methyl stearate	39.5	9.0–20.9	HTF in a GCHP
[138]	num	finite element model	V	paraffin	/	190	GCHP borehole
[139]	num	three-dimensional unsteady model	V	micro-encapsulated paraffin. Shape-stabilized decanoic and lauric acid mixture	23–27 19.9	150 109.2	grout with a GCHP
[132]	exp & num	three-dimensional computational fluid dynamics	H	decyl acid and lauric acid (66:34) oleic acid	20.55 8.11	133.65 94.51	backfilling material of a GHE
[129]	num	three-dimensional computational fluid dynamics	H	salt hydrate (Infinite R, Insolcorp)	23	200	UTB with GCHP
[135]	num	Eulerian–Eulerian approach	H	micro-encapsulated n-octadecane	28–30	167	HTF in a GCHP
[130]	exp & num	computational fluid dynamics	V	RT6 RT27	8.0–8.5 25.0–25.5	140 146	integrated with a GCHP
[133]	exp	/	H	n-octadecane paraffin A28	28 28	241 265	backfilling material of a GCHP

From these results, it is clear that very different ways of enhancing GCHPs’ performances and issues through PCMs have been tested, but current studies suffer from a lack of experimental data and validation, as almost all of them present only numerical analyses. Another important aspect is the system’s end-of-life, as PCM recovery and disposal would be easier for horizontal GCHPs compared to vertical GCHPs.

Another possible use of PCMs concerns energy piles technology, where a closed loop consisting of a GHE integrated into the concrete of foundation piles of structures can be exploited [140]. Although several studies have already analyzed this technology, energy piles still face different critical challenges, such as the effect of temperature variations, due to the GCHP, on the internal stress distribution of the concrete pile or its bearing capacity [141].

In addition, the integration of PCMs into concrete would enhance the GCHP’s efficiency. However, there are many challenges linked to interactions between concrete, GHE pipes, and PCMs, while the number of related studies is still very limited [142,143]. The integration between PCMs and backfilling materials, or concrete in energy piles, could also take advantage of previous studies performed in the building sector. For instance, studies on the integration of PCMs in envelope materials as mortars or concrete (Table 7) may be a good starting point to create backfilling grouts with known thermal and mechanical properties. However, GHE backfilling contributes to the building’s footprint. Therefore, its enhancement should be considered as a further strategic opportunity for the integration of larger thermal energy storage, which can better mitigate the synchrony loss between supply and demand in using renewable energies.

Furthermore, the integration of PCMs with GHE backfilling materials does not affect the building volume, (as occurs with HVAC), and the lower digging cost compensates for its installation. Recently, to further reduce the GCHPs’ installation costs by shortening the GHEs’ length, multi-source heat pump systems have been studied and designed on a residential scale and for small building volumes [144,145]. The focus is on the exploitation of the most advantageous energy source (air, ground, solar), which is selected according to evolved temperatures. However, the ground is the sole source able to carry out thermal storage and can therefore play a pivot role, which may be improved by PCMs.

## 4. Conclusions

This work analyzed the main review and experimental studies concerning the integration of phase change materials into building applications during the last twenty years. The evolution of the research in this field was divided into three main stages (2000–2010, 2011–2015, and 2016–2021), and the evolution concerning phase change materials and their application was discussed, with a focus on the studies performed between 2019 and 2021. Some conclusions can be drawn:

- among different classes of PCM, paraffins are generally preferred for integration in building materials, due to their high performance/cost ratio, but more recent studies (2019–2021) also consider other organic PCMs, namely alcohols and fatty acids, as more specific applications need tailored materials.
- microencapsulation and shape stabilization represent the main technologies actually used to incorporate PCMs into building materials, namely wallboards, mortars, and concrete, as they ensure better thermal properties, while macro-encapsulation is preferred for glazing elements, due to their higher chemical stability.
- Field- and laboratory-scale tests designed to evaluate the influence of PCMs on indoor thermal conditions are mainly focused on passive conditions and on avoiding active HVAC plants. Nevertheless, the generally inconstant performance of PCMs due to climate or seasonal conditions was determined. The use of active heating/cooling systems, based on PCM employment, is highly recommended, ensuring a constant performance of building elements during the same season or the whole year.

Finally, an overview of the integration of PCMs with ground-coupled heat pump (GCHP) technology was carried out. The integration between PCM and ground-coupled heat pump technologies is still at an early stage, as almost all the relevant studies are performed only at the numerical level. The use of PCMs seems to be very promising for horizontal GCHPs, as it allows the stabilization of soil temperature fluctuations and, consequently, the whole performance, without high installation costs.

However, future research related to this new technology could greatly benefit from the outcomes of previous studies about the integration of PCMs in building materials. The integration of PCMs in the backfilling material could take advantage of the studies on the integration of PCM in mortars and concrete. These materials are useful as backfilling for GCHPs, as there are no requirements connected to their high mechanical properties. The use of natural materials, such as expanded perlite or vermiculite, and their integration with paraffins or biodegradable PCMs (such as PureTemp 23 or coconut oil) could also help the development of this technology in a more environmentally friendly way, avoiding potential environmental problems such as the leakage of melted PCMs into the ground.

## Figures and Tables

**Table 1:** Principal advantages and disadvantages of organic, inorganic, and eutectic PCMs [15].

	Organics	Inorganics	Eutectics
Advantages	Non-corrosive Low or no undercooling Chemical and thermal stability	Greater phase change enthalpy Greater density	Sharp melting point
Disadvantages	Lower phase change enthalpy Low thermal conductivity Flammability	Undercooling	Lack of data
		Corrosion	
		Phase separation	
		Phase segregation, lack of thermal stability	

**Table 2:** PCM classification according to different application types and temperature range in the building sector up to 2010, according to Cabeza et al. [43].

	Cooling	Comfort Applications	Hot-Water Applications	High-Temperature Applications
Temperature range	−30/+21 °C	+22/+28 °C	+29/+60 °C	+61/+120 °C
Total PCM number	45	34	103	62
Organic (paraffins, fatty acids, organic mixtures) (%)	23 (51.1%)	22 (64.7%)	50 (48.5%)	28 (45.2%)
Inorganic (salt, salt hydrates, metals, inorganic mixtures) (%)	6 (13.3%)	7 (20.6%)	45 (43.7%)	19 (30.6%)
Eutectics (%)	14 (31.1%)	5 (14.7%)	8 (7.8%)	15 (24.2%)

**Table 3:** Classification of commercial PCMs according to different application types and temperature ranges in the building sector up to 2010, according to Cabeza et al. [43].

	Cooling	Comfort Applications	Higher-Temperature Applications
Temperature range	−33/+21 °C	+22/+28 °C	≥29 °C
Total PCM number	24	13	51
Organic (paraffins) (%)	2 (8.3%)	5 (38.5%)	29 (56.9%)
Inorganic (salt solutions, salt hydrates) (%)	22 (91.7%)	8 (61.5%)	20 (39.2%)
Unclassified (%)	0 (0.0%)	0 (0.0%)	2 (3.9%)

**Table 4:** PCMs studied for building applications in the period 2016–2021, according to Akeiber et al. [19], Liu et al. [56], Singh Rathore et al. [57], and Da Cunha and De Aguiar [58].

	Category	Melting Point (°C)	Encapsulation Type	Shell Material	Reference
RT 18	Organic	18	Macro	Steel	[56]
Capric acid and lauric acid	Fatty acids	20	Macro	Stainless steel	[56,58]
RT 21	Paraffin	21	Macro	EPDM and furnace dust	[19]
SP 22	Inorganic	21	Macro	/	[19]
Hexadecane	Paraffin	22	Macro	Copper	[56]
Micronal DS-5008X	Organic	23	Micro	Acrylate polymer	[19,57,58]
Inertek	Organic	23+27	Micro	Polymer	[58]
PEG 600	Polymer	21–25	Macro	PVC	[56,57,58]
Micronal DS 5001 X	Organic	23–26	Micro	Acrylate polymer	[57,58]
Capric acid and 1-dodecanol	Fatty acid and fatty alcohol	26	Macro	Aluminum	[19,56]
Dodecanol	Fatty alcohol	26	Micro	/	[57]
Capric acid and myristic acid	Fatty acids	26	Micro	Polystyrene	[57]
Capric acid and palmitic acid	Fatty acids	26	Macro	Gypsum wallboard	[19,56]
SP 25	Salt hydrate	26	Macro	Aluminum	[56,58]
Calcium chloride hexahydrate	Salt hydrate	25–27	Macro	PVC	[19,56]
RT 27	Organic	28	Macro Micro	Aluminum /	[56,57,58]
RT 28	Organic	28	Micro	/	[57]
RT 28HC	Organic	27–29	Macro	Aluminum	[19,56,58]
MG29	Paraffin	27–29	Macro	Glass	[19]
Octadecane	Paraffin	28–29	Micro Micro	CaCl_2_ PMMA/TiO_2_	[57]
Eicosane	Paraffin	30	Micro Macro	Brookite (TiO_2_) Galvanized steel	[57,58]
Capric Acid	Fatty acid	30	Macro	Aluminum	[19,56]
Salt hydrate	Salt hydrate	31	Macro	Polymer	[56]
RT 35	Organic	28–35	Macro	Aluminum	[19,56]
Tetradecanol and myristic acid	Fatty alcohol and fatty acid	29–32	Macro	PE-RT	[56]
MPCM37-D	Paraffin	37	Micro	Polymer	[57]
RT 42	Organic	38–43	Macro	Stainless steel	[56]

**Table 5:** Main experimental studies on passive applications performed on building elements containing PCMs between 2019 and 2021.

Reference	Test Scale	PCM	PCM Form	T_melt_ (°C)	Building Material	Position in Building	Remarks and Results
[61]	field test	RT28HC	macro	28	aluminum panel	wall	The PCM decreased the cooling load, peak, and average temperature in the room by 0.8 °C when coupled with the radiative panel.
[62]	field test	PX 35	micro	35	aluminum panel	blind in a double-skin façade	The PCM blind was used to reduce the overheating problem typical of double skin facades in summer, stabilizing the internal air temperature between the two glass layers.
[63]	field test	paraffin	SSPCM (paraffin + graphite)	25.5	concrete panel	roof	The PCM roof allowed a reduction of indoor air temperature fluctuations from 7% to 15%. Combined with a high-reflectivity roof, it reduced the indoor air temperature fluctuation from 8.5% to 17.0%, while the inner surface temperature of the roof was reduced by 2.2 °C.
[64]	lab test	paraffins	SSPCM	25, 31 and 44	polyurethane membrane	roof	The integration of PCMs reduced both indoor and materials’ temperature. Cool roof materials benefit from lower phase change temperatures (25–35 °C), while common dark membranes show a better performance with higher temperatures (31–45 °C).
[65]	lab test	CaCl_2_ ∗ 6H_2_O	macro (Polyvinyl chloride)	26	wallboard	Wall	PCM reduced the indoor average temperature and its fluctuations, but this was highly dependent on climate conditions.
[66]	lab and field test	paraffin	nano	27.4	wood fiber-polymer composite	floor	Tensile strength of the wood fiber composite was reduced up to 58%, and flexural strength up to 68% with 40 wt% of PCM. Use of PCM coupled with natural night ventilation help to reduce the overheating period (temperatures above 23 °C) to about 50%.
[67]	field test	coconut oil	macro	22.6	pouches	walls, windows	The PCM in south-facing walls and window allowed a higher reduction of indoor temperatures (up to 7.2 °C) If the PCM is applied only on the wall, the reduction is equal to 5.2 °C.
[68]	field test	n-octadecane	nano	23.3	plaster wallboard	walls, ceiling	The PCM (30 wt%) helped to stabilize the indoor temperature but not enough to reach the comfort range (18–23 °C), so natural night-time ventilation is required during summer.
[69]	field test	RT28	macro	27.5	integration of separate TES in the window	window	The PCM enhanced the heat balance of the window by 10% during the heating season but suffered from overheating during summer.
[70]	field test	CaCl_2_ ∗ 6H_2_O and MgCl ∗ 6H_2_O mixture	macro	21	polyethylene containers	wall	The PCM integration in the wood walls showed a reduction in overall temperature fluctuations equal to 57%, while the day/night fluctuations were reduced by 62% with respect to the reference room.
[71]	field test	1-tetradecanol 1-hexadecanol	SSPCM (PCM + diatomite)	33.8 (tetradecanol) 41.8 (hexadecanol)	plaster wallboard	wall	Tetradecanol attenuated better than hexadecanol heat waves at the west wall. However, the reference board performed better on both PCM-enhanced boards when placed on the east wall. This suggests that the suitable phase change temperature depends also on the orientation of the elements of the building.
[72]	lab test	n-octadecane (RT26)	macro	32.2	copper or PVC tubes embebbed in insulated panels	wall	Depending on the encapsulating material and on the orientation of capsules (vertical/horizontal), the PCM (12–15%) reduced the peak heat flux in the wall between 12% and 33% with respect to a reference insulation panel. Copper was shown to be more efficient than PVC as encapsulating material.
[73]	field test	RT28HC	macro	28	glass	window	The internal temperature of the window decreased by 7.6 °C when filled with PCM instead of air, and the CFD model suggested that PCM thickness should not exceed 30 mm.

**Table 6:** Main experimental studies on active applications performed on building elements containing PCMs between 2019 and 2021.

Reference	Test Scale	PCM	PCM Form	T_melt_ (°C)	Building Material	Position in Building	Remarks and Results
[74]	field test	paraffin (DuPont Energain). Soy and palm oil (BioPCM Q25 M51)	SSPCM (DuPont Energain) macro (BioPCM)	21.6 (Energain) 25 (BioPCM)	wallboard laminated with aluminum (Energain) pouches (BioPCM)	walls, ceiling	BioPCM was not efficient for cooling during summer, but it was found to be effective in winter, to increase the thermal inertia of buildings. Energain reduced the peak temperature by 3–4 °C and the daily temperature fluctuations to 1–2 °C.
[75]	lab test	paraffin	SSPCM	17.2	resin sheet	floor	The PCM increased maximum floor temperatures during heating and cooling respectively by 5.04 °C and 1.08 °C. The cooling delay time of the floor was increased up to 3.6 h.
[76]	lab test	paraffin	macro	24	Panel steel	ceiling	Macroencapsulated PCM showed higher cooling power with respect to the one with microencapsulated PCM but lower respect to the one without PCM. This panel also showed greater flexibility in shifting the cooling load to off-peak hours.
[77]	field test	coconut oil	macro	25	steel	floor, wall, ceiling	The inclusion of PCM was useful to shift the heating load to low-peak periods. Inclusion of PCM in the ceiling was less efficient than inclusion in floor and walls.
[78]	field test	not specified	micro	27.8	steel	floor	The PCM increased the thermal storage capacity of the floor by 77.36% and stabilized indoor thermal stability. The heat gained from solar radiation and stored in the PCM floor can increase indoor air temperature by 3 °C if coupled with the ventilation system.

**Table 7:** Experimental studies on the inclusion of PCMs in concrete and mortars between 2019 and 2021.

Reference	PCM	PCM Form	PCM T_melt_ (°C)	Supporting Material	Building Material	Incorporation in the Concrete/Mortar	PCM Latent Heat Capacity (kJ/kg)	Results
[81]	Butyl stearate	bulk	23.4	none	concrete	direct	134.2	The PCM prevented a concrete temperature rise, improved its workability, and reduced the corrosive damages on steel embedded in concrete.
[84]	PEG 1000	SSPCM	37–40	Lecce stone	Hydraulic lime, gypsum, cement mortars	impregnated aggregates	129	The PCM lowered both the phase change temperatures (from 37–40 °C to 13–17 °C) and the phase change enthalpy (from 129 kJ/kg to 7–9 kJ/kg). Both flexural and compressive strength showed a considerable decrease for all the binders with a water increment of 15%.
[85]	Micronal 5008 (Octadecane)	micro	23	acrylic polymer	Alkali-activated cements	microcapsules	100	Microencapsulated PCM (up to 20%) enhanced the heat storage capacity of the cement but decreased its compressive strength from 43% to 50%.
[90]	Capric acid and myristyl alcohol (weight ratio = 9:1)	SSPCM	17–32	expanded perlite	cement	impregnated aggregates	167.2	The addition of composite PCM to the cement led to a significant decrease in its compressive strength (from 54% to 82% decrease for 10 wt% to 30 wt% of composite addition). Indoor temperature fluctuations were reduced, and no leakage was shown.
[88]	Butyl stearate	macro	19	steel balls	concrete	blending of macrocapsules and concrete	107.3	The use of steel balls (30%) prevented leakage problems but reduced the concrete’s compressive strength by 18%. A total of 5% of the cement mass was replaced with slag and fly ash to solve this problem. Coarse aggregates substitution (10% in volume) with steel balls greatly enhanced the heat transfer efficiency without lowering too much the mechanical properties.
[82]	paraffin	bulk	20–23	none	concrete	direct	107.3	PCM addition to concrete reduced compressive strength from 18.5 MPa (0% PCM) to 14.9 MPa (20% PCM). The addition of PCM up to 10% does not cause significant changes in flexural and compressive strength. The direct incorporation of PCM increased the liquid/binder ratio and decreased the water absorption of concrete.
[91]	BSF26	micro	26	not specified	Alkali-activated cements	blending of microcapsules and cement	110	The addition of PCM from 0% to 30% caused a serious reduction in mechanical properties (compressive and flexural strength). No more than 20% of PCM should be added.
[83]	1-dodecanol	bulk	22	none	Portland cement	direct	195	The mortar was enriched with 6 wt% of PCM, enhanced with copper oxide and titania, generating a 10% decrease in the compressive strength
[92]	octadecane (75%) and eicosane (25%) eutectic mixture	SSPCM	20.4	pumice	cement plaster	impregnated aggregates	232.7	SSPCM (Pumice + 34 wt% of PCM) showed good compatibility, no leakage, and good thermal stability. Cement mortar was formed with 30 wt% of the SSPCM achieving sufficient thermal regulation properties.
[93]	capric acid and polyethylene glycol (PEG 600)	SSPCM	31.3 (Capric acid) 9.9 (PEG)	pumice	Portland cement	impregnated aggregates	190.2 (Capric acid) 148.5 (PEG)	The SSPCMs (62 wt% of capric acid and 56 wt% of PEG) showed good thermal stability. Plaster enriched with SSPCM (20 wt%) for thermal regulation in buildings.
[94]	PureTemp 23		23	expanded perlite, hydrated lime	hydraulic lime and Portland cement plaster	impregnated materials	227	A plaster with 6 wt% PCM was tested, showing no leakage problems and compressive strength, similar to a commercial plaster taken as reference.
[95]	n-nonacosane	SSPCM	64	expanded perlite	Portland cement concrete	impregnated aggregates	195	No leakage was detected and the time delay in temperature rising was verified with respect to concrete without PCM. Compressive strength decreased by 40% (from 37.5 MPa to 22.5 MPa).
[86]	Lauric acid (66 wt%) and myristic acid (34 wt%) eutectic mixture	SSPCM	32.2	fly ash	Portland cement mortar	impregnated fly ash	177	The addition of 20 wt% of SSPCM (37% of PCM) to the cement mortar reduced the compressive strength by 54% (from 45.12 MPa to 20.21 MPa) and flexural strength by 67% (from 5.25 MPa to 1.74 MPa).
[87]	Capric acid (82 wt%) and stearic acid (18 wt%) eutectic mixture	SSPCM	24.7	silica fume	Portland cement mortar	impregnated silica fume	178	The addition of 20% of SSPCM (27% of PCM) to the mortar showed good temperature regulation properties. Its compressive and flexural strength were respectively decreased by 37% and 36.57% compared with the reference mortar.
[96]	Nextek 37D	micro	37	not specified	polymer modified cement mortar	mechanical blending	190	Up to 20 wt% of PCM was added to the mortar. The compressive strength decreased from 64 MPa to 14 MPa, while the flexural strength decreased from 8.6 MPa to 4.8 MPa.
[97]	RT27 (paraffin)	SSPCM	26.5	expanded perlite	geopolymer concrete, geopolymer foam concrete	impregnated materials	189	The addition of 15 wt% and 30 wt% of SSPCM decreased the compressive strength of the geopolymer concrete, respectively, by 35% and 64%. However, the addition of 30 wt% SSPCM to the geopolymer foam concrete enhanced its compressive strength by 87% and its thermal storage capacity by 181%.
[98]	paraffin	SSPCM	58.1	clasting light shale ceramsite	Portland cement concrete	impregnated aggregates	178.3	Up to 6 wt% of PCM was added to the concrete. The compressive strength decreased by 76.5% compared with the reference concrete, while specific heat capacity increased by 41.2%.
[89]	paraffin	SSPCM	25.2	expanded vermiculite and diatomite	Portland cement	impregnated aggregates	175.6	The use of diatomite increased the thermal storage capacity of the SSPCM (52 wt% of PCM) by 15.6% and enhanced both its strength and long-term stability. The mortar with diatomite-based SSPCM had a compressive strength 25% higher with respect to the mortar enriched only with vermiculite-based PCM (45 wt%).

**Table 8:** Environmental impact of PCMs for building applications. Adaptation from Kylili and Fokaides [18,101,102].

PCM	Name in the EI99	Impact kg/Material [EI99 Pt]
Paraffin	Paraffin, at plant, RER	0.208
Salts hydrates	Calcium chloride, CaCl_2_, at regional storage, CH	0.058
Disposal, paraffin	(assumption)	0.015
Disposal, salts hydrates	(assumption)	0.008

**Table 9:** Experimental studies on the use of natural substances or wastes as PCMs or supporting materials.

Reference	PCM	PCM T_melt_ (°C)	Supporting Material	Type of Incorporation	Composite Latent Heat Capacity (kJ/kg)	Remarks and Results
[105]	capric acid, lauric acid	25.5	fly ash	vacuum adsorption	45.38	Fly ash was obtained from a power plant.
[106]	PEG 1000	38	transparent (delignified) wood and polymethyl methacrylate	vacuum impregnation	76	The composite material showed good transmittance up to 84% by decreasing thickness (up to 0.5 mm) of the composite material. No changes in elastic modulus were observed, except a reduction in flexural strength (70.5 MPa instead of 129.6 MPa) due to the inclusion of PCM.
[107]	n-heptadecane	25.1	activated carbon from pine cones	one-step impregnation	138.2	Heptadecane 62 wt% was found to be the optimum content, to avoid leakage and enhance thermal conductivity.
[108]	paraffin	52.1	rice husk ash	mechanical mixing and impregnation	68.1	A paraffin/rice husk ratio equal to 50% prevented leakage problems. PCM and rice husk ash showed good compatibility and thermal stability.
[109]	capric acid (83 wt%) and stearic acid (17 wt%) eutectic mixture	24.7	Scots pine sapwood	vacuum impregnation	94	The composite showed good chemical and thermal performance stability after 600 phase change cycles. The presence of PCMs decreased the water absorption from 80% to 20%, enhancing wood’s hydrophobicity and anti-swelling efficiency. The mechanical properties of wood were also enhanced: modulus of rupture (+22.3%), modulus of elasticity (25.3%), and compression strength parallel to grain (24.5%).
[110]	NaHPO_4_ ∗ 12H_2_O (58 wt%) and Na_2_CO_3_ ∗ 10H_2_O (42 wt%) eutectic mixture	25	diatomite, polyurethane acrylate	impregnation, coating, UV curing	102.6	The composite material, with 40% of diatomite, was coated with a polymer to avoid leakage problems. Supercooling was almost eliminated (0.5 °C) performance stability confirmed up to 300 phase change cycles.
[111]	Nextek 24D (paraffin and polymeric shell)	22.4	silty-clay soil and reed fiber	mechanical mixing	Not specified	A microencapsulated PCM was integrated (up to 20 wt%) in a soil and reed fiber mixture. The thermal conductivity decreased by up to 14%. Water vapor permeability’s decrease was 20%. The compressive strength was not affected by the addition of PCM; however, the soil-fiber mixture itself showed low values of compressive strength.
[112]	PureTemp 23	23	cuttlebone pomelo peel	one-step impregnation	145 for both composites	PCM and supporting materials are both biodegradable and obtained from renewable sources. Good chemical compatibility and limited leakage was demonstrated with a thermal storage efficiency equal to 70% of the pure PCM. The performance stability was confirmed up to 100 cycles.
[113]	organic	29.9	porcelain stoneware and soda-lime glass	vacuum impregnation	Not specified	The PCM impregnation efficiency in the glass-ceramic foam was between 24% and 39%. Thermal properties still need to be measured and leakage problems need to be addressed.
[114]	OM37 (inorganic)	39.1	expanded graphite and expanded vermiculite	ultrasonication and vacuum impregnation	99.3	The addition of expanded graphite up to 7% led to a decrease in latent heat storage capacity, while thermal conductivity increased by 114% and no leakage was detected.
[115]	PureTemp 23	201	expanded glass aggregates and fly ash (coating)	vacuum impregnation	92.7	Glass aggregates absorbed up to 80% of PCM and, when coated with fly ash, showed no leakage problems.

## Data Availability

Not applicable.

## References

[1] United Nations Environment Programme (UNEP), International Energy Agency (IEA) (2017). Towards a Zero-Emission, Efficient, and Resilient Buildings and Construction Sector.

[2] International Energy Agency (IEA) (2020). Tracking Buildings 2020.

[3] European Parliament (2018). Directive (EU) 2018/844 of the European Parliament and of the Council of 30 May 2018 amending. Off. J. Eur. Union.

[4] European Parliament (2010). Directive 2010/31/EU of the European Parliament and of the Council of 19 May 2010 on the Energy Performance of Buildings. Off. J. Eur. Union.

[5] Stritih U., Tyagi V.V., Stropnik R., Paksoy H., Haghighat F., Joybari M.M. (2018). Integration of passive PCM technologies for net-zero energy buildings. Sustain. Cities Soc..

[6] Sharma A., Tyagi V.V., Chen C.R., Buddhi D. (2009). Review on thermal energy storage with phase change materials and applications, Renew. Sustain. Energy Rev..

[7] Jemmal Y., Zari N., Maaroufi M. (2016). Thermophysical and chemical analysis of gneiss rock as low cost candidate material for thermal energy storage in concentrated solar power plants. Sol. Energy Mater. Sol. Cells.

[8] Rathore P.K.S., Shukla S.K. (2019). Potential of macroencapsulated PCM for thermal energy storage in buildings: A comprehensive review. Constr. Build. Mater..

[9] Baetens R., Jelle B.P., Gustavsen A. (2010). Phase change materials for building applications: A state-of-the-art review. Energy Build..

[10] Osterman E., Tyagi V.V., Butala V., Rahim N.A., Stritih U. (2012). Review of PCM based cooling technologies for buildings. Energy Build..

[11] Kenisarin M., Mahkamov K. (2015). Salt hydrates as latent heat storage materials: Thermophysical properties and costs. Sol. Energy Mater. Sol. Cells.

[12] Wong-Pinto L.-S., Milian Y., Ushak S. (2020). Progress on use of nanoparticles in salt hydrates as phase change materials. Renew. Sustain. Energy Rev..

[13] Lin Y., Alva G., Fang G. (2018). Review on thermal performances and applications of thermal energy storage systems with inorganic phase change materials. Energy.

[14] Farid M.M., Khudhair A.M., Razack S.A.K., Al-Hallaj S. (2004). A review on phase change energy storage: Materials and applications. Energy Convers. Manag..

[15] Mohamed S.A., Al-Sulaiman F.A., Ibrahim N.I., Zahir H.M., Al-Ahmed A., Saidur R., Yılbaş B.S., Sahin A.Z. (2017). A review on current status and challenges of inorganic phase change materials for thermal energy storage systems. Renew. Sustain. Energy Rev..

[16] Nazir H., Batool M., Bolivar Osorio F.J., Isaza-Ruiz M., Xu X., Vignarooban K., Phelan P., Inamuddin, Kannan A.M. (2019). Recent developments in phase change materials for energy storage applications: A review. Int. J. Heat Mass Transf..

[17] Fallahi A., Guldentops G., Tao M., Granados-Focil S., Van Dessel S. (2017). Review on solid-solid phase change materials for thermal energy storage: Molecular structure and thermal properties. Appl. Therm. Eng..

[18] de Gracia A., Cabeza L.F. (2015). Phase change materials and thermal energy storage for buildings. Energy Build..

[19] Akeiber H., Nejat P., Majid M.Z.A., Wahid M.A., Jomehzadeh F., Zeynali Famileh I., Calautit J.K., Hughes B.R., Zaki S.A. (2016). A review on phase change material (PCM) for sustainable passive cooling in building envelopes. Renew. Sustain. Energy Rev..

[20] Iten M., Liu S., Shukla A. (2016). A review on the air-PCM-TES application for free cooling and heating in the buildings. Renew. Sustain. Energy Rev..

[21] Aranda-Usón A., Ferreira G., López-Sabirón A.M., Mainar-Toledo M.D., Zabalza Bribián I. (2013). Phase change material applications in buildings: An environmental assessment for some Spanish climate severities. Sci. Total Environ..

[22] de Gracia A., Rincón L., Castell A., Jimenez M., Boer D., Medrano M., Cabeza L.F. (2010). Life Cycle Assessment of the inclusion of phase change materials (PCM) in experimental buildings. Energy Build..

[23] Baldassarri C., Sala S., Caverzan A., Lomperti Tornaghi M. (2017). Environmental and spatial assessment for the ecodesign of a cladding system with embedded Phase Change Materials. Energy Build..

[24] Nienborg B., Gschwander S., Munz G., Fröhlich D., Helling T., Horn R., Weinläder H., Klinker F., Schossig P. (2018). Life Cycle Assessment of thermal energy storage materials and components. Energy Procedia.

[25] Sutterlin W.R. Phase Change Materials: A Brief Comparison of Ice Packs, Salts, Paraffins, and Vegetable-Derived Phase Change Materials—Pharmaceutical Outsourcing. https://www.pharmoutsourcing.com/Featured-Articles/37854-Phase-Change-Materials-A-Brief-Comparison-of-Ice-Packs-Salts-Paraffins-and-Vegetable-derived-Phase-Change-Materials/.

[26] Zalba B., Marín J.M., Cabeza L.F., Mehling H. (2003). Review on thermal energy storage with phase change: Materials, heat transfer analysis and applications. Appl. Therm. Eng..

[27] Verma P., Varun, Singal S. (2008). Review of mathematical modeling on latent heat thermal energy storage systems using phase-change material. Renew. Sustain. Energy Rev..

[28] Regin A.F., Solanki S., Saini J. (2008). Heat transfer characteristics of thermal energy storage system using PCM capsules: A review. Renew. Sustain. Energy Rev..

[29] Jegadheeswaran S., Pohekar S.D. (2009). Performance enhancement in latent heat thermal storage system: A review. Renew. Sustain. Energy Rev..

[30] Tyagi V.V., Buddhi D. (2007). PCM thermal storage in buildings: A state of art. Renew. Sustain. Energy Rev..

[31] Chekhovskoi V.Y. (2000). Thermal expansion and density of 80.5% LiF-19.5% CaF_2_ eutectic. High Temp..

[32] Agyenim F., Hewitt N., Eames P., Smyth M. (2010). A review of materials, heat transfer and phase change problem formulation for latent heat thermal energy storage systems (LHTESS). Renew. Sustain. Energy Rev..

[33] Raj V.A.A., Velraj R. (2010). Review on free cooling of buildings using phase change materials. Renew. Sustain. Energy Rev..

[34] Zalba B., Marín J.M., Cabeza L.F., Mehling H. (2004). Free-cooling of buildings with phase change materials. Int. J. Refrig..

[35] Zalba B.C.L., Marin J.M., Sanchez-Valverde B. Free cooling: An application of PCMS in TES. Proceedings of the 3rd Workshop IEA ECES IA (Annex 17).

[36] Lazaro A., Dolado P., Marín J.M., Zalba B. (2009). PCM–air heat exchangers for free-cooling applications in buildings: Experimental results of two real-scale prototypes. Energy Convers. Manag..

[37] Agyenim F., Eames P., Smyth M. (2010). Heat transfer enhancement in medium temperature thermal energy storage system using a multitube heat transfer array. Renew. Energy.

[38] Papanicolaou E., Belessiotis V. (2002). Transient natural convection in a cylindrical enclosure at high Rayleigh numbers. Int. J. Heat Mass Transf..

[39] Zhang P., Ma Z., Wang R. (2010). An overview of phase change material slurries: MPCS and CHS. Renew. Sustain. Energy Rev..

[40] Lacroix M. (1993). Study of the heat transfer behavior of a latent heat thermal energy storage unit with a finned tube. Int. J. Heat Mass Transf..

[41] Jegadheeswaran S., Pohekar S., Kousksou T. (2010). Exergy based performance evaluation of latent heat thermal storage system: A review. Renew. Sustain. Energy Rev..

[42] Lock G.S. (1994). Latent Heat Transfer.

[43] Cabeza L.F., Castell A., Barreneche C., De Gracia A., Fernández A.I. (2011). Materials used as PCM in thermal energy storage in buildings: A review. Renew. Sustain. Energy Rev..

[44] Zhou D., Zhao C.Y., Tian Y. (2012). Review on thermal energy storage with phase change materials (PCMs) in building applications. Appl. Energy.

[45] Kalnæs S.E., Jelle B.P. (2015). Phase change materials and products for building applications: A state-of-the-art review and future research opportunities. Energy Build..

[46] Rathod M.K., Banerjee J. (2013). Thermal stability of phase change materials used in latent heat energy storage systems: A review. Renew. Sustain. Energy Rev..

[47] Konuklu Y., Ostry M., Paksoy H.O., Charvat P. (2015). Review on using microencapsulated phase change materials (PCM) in building applications. Energy Build..

[48] Cao L., Su D., Tang Y., Fang G., Tang F. (2015). Properties evaluation and applications of thermal energystorage materials in buildings. Renew. Sustain. Energy Rev..

[49] Pomianowski M., Heiselberg P., Zhang Y. (2013). Review of thermal energy storage technologies based on PCM application in buildings. Energy Build..

[50] Soares N., Costa J.J., Gaspar A.R., Santos P. (2013). Review of passive PCM latent heat thermal energy storage systems towards buildings’ energy efficiency. Energy Build..

[51] Memon S.A. (2014). Phase change materials integrated in building walls: A state of the art review. Renew. Sustain. Energy Rev..

[52] Kuznik F., David D., Johannes K., Roux J.-J. (2011). A review on phase change materials integrated in building walls. Renew. Sustain. Energy Rev..

[53] Ling T.C., Poon C.S. (2013). Use of phase change materials for thermal energy storage in concrete: An overview. Constr. Build. Mater..

[54] Castell A., Menoufi K., de Gracia A., Rincón L., Boer D., Cabeza L.F. (2013). Life Cycle Assessment of alveolar brick construction system incorporating phase change materials (PCMs). Appl. Energy.

[55] Menoufi K., Castell A., Farid M., Boer D., Cabeza L.F. (2013). Life Cycle Assessment of experimental cubicles including PCM manufactured from natural resources (esters): A theoretical study. Renew. Energy.

[56] Liu Z., Yu Z., Yang T., Qin D., Li S., Zhang G., Haghighat F., Joybari M.M. (2018). A review on macro-encapsulated phase change material for building envelope applications. Build. Environ..

[57] Singh Rathore P.K., Shukla S.K., Gupta N.K. (2020). Potential of microencapsulated PCM for energy savings in buildings: A critical review. Sustain. Cities Soc..

[58] da Cunha S.R.L., de Aguiar J.L.B. (2020). Phase change materials and energy efficiency of buildings: A review of knowledge. J. Energy Storage.

[59] Song M., Niu F., Mao N., Hu Y., Deng S.S. (2018). Review on building energy performance improvement using phase change materials. Energy Build..

[60] Zhu N., Li S., Hu P., Wei S., Deng R., Lei F. (2018). A review on applications of shape-stabilized phase change materials embedded in building enclosure in recent ten years. Sustain. Cities Soc..

[61] He W., Yu C., Yang J., Yu B., Hu Z., Shen D., Liu X., Qin M., Chen H. (2019). Experimental study on the performance of a novel RC-PCM-wall. Energy Build..

[62] Li Y., Darkwa J., Kokogiannakis G., Su W. (2019). Phase change material blind system for double skin façade integration: System development and thermal performance evaluation. Appl. Energy.

[63] Meng E., Wang J., Yu H., Cai R., Chen Y., Zhou B. (2019). Experimental study of the thermal protection performance of the high reflectivity-phase change material (PCM) roof in summer. Build. Environ..

[64] Piselli C., Castaldo V.L., Pisello A.L. (2019). How to enhance thermal energy storage effect of PCM in roofs with varying solar reflectance: Experimental and numerical assessment of a new roof system for passive cooling in different climate conditions. Sol. Energy.

[65] Qiao Y., Yang L., Bao J., Liu Y., Liu J. (2019). Reduced-scale experiments on the thermal performance of phase change material wallboard in different climate conditions. Build. Environ..

[66] Valizadeh S., Ehsani M., Torabi Angji M. (2019). Development and thermal performance of wood-HPDE- PCM nanocapsule floor for passive cooling in building. Energy Sources Part A Recovery Util. Environ. Eff..

[67] Alqahtani T., Mellouli S., Bamasag A., Askri F., Phelan P.E. (2020). Experimental and numerical assessment of using coconut oil as a phase-change material for unconditioned buildings. Int. J. Energy Res..

[68] Maleki B., Khadang A., Maddah H., Alizadeh M., Kazemian A., Ali H.M. (2020). Development and thermal performance of nanoencapsulated PCM/ plaster wallboard for thermal energy storage in buildings. J. Build. Eng..

[69] Musiał M. (2020). Experimental and Numerical Analysis of the Energy Efficiency of Transparent Partitions with a Thermal Storage Unit. J. Ecol. Eng..

[70] Sonnick S., Erlbeck L., Gaedtke M., Wunder F., Mayer C., Krause M.J., Nirschl H., Rädle M. (2020). Passive room conditioning using phase change materials—Demonstration of a long-term real size experiment. Int. J. Energy Res..

[71] Yu H., Li C., Zhang K., Tang Y., Song Y., Wang M. (2020). Preparation and thermophysical performance of diatomite-based composite PCM wallboard for thermal energy storage in buildings. J. Build. Eng..

[72] Zhang Y., Sun X., Medina M.A. (2020). Experimental evaluation of structural insulated panels outfitted with phase change materials. Appl. Therm. Eng..

[73] Bolteya A.M., Elsayad M.A., Belal A.M. (2021). Thermal efficiency of PCM filled double glazing units in Egypt. Ain Shams Eng. J..

[74] Sinka M., Bajare D., Jakovics A., Ratnieks J., Gendelis S., Tihana J. (2019). Experimental testing of phase change materials in a warm-summer humid continental climate. Energy Build..

[75] Yun B.Y., Yang S., Cho H.M., Wi S., Kim S. (2019). Thermal Storage Effect Analysis of Floor Heating Systems Using Latent Heat Storage Sheets. Int. J. Precis. Eng. Manuf. Green Technol..

[76] Bogatu D.I., Kazanci O.B., Olesen B.W. (2021). An experimental study of the active cooling performance of a novel radiant ceiling panel containing phase change material (PCM). Energy Build..

[77] Faraj K., Khaled M., Faraj J., Hachem F., Castelain C. (2021). Experimental Study on the Use of Enhanced Coconut Oil and Paraffin Wax Phase Change Material in Active Heating Using Advanced Modular Prototype. J. Energy Storage.

[78] Guo J., Dong J., Wang H., Jiang Y., Tao J. (2021). On-site measurement of the thermal performance of a novel ventilated thermal storage heating floor in a nearly zero energy building. Build. Environ..

[79] Rao V.V., Parameshwaran R., Ram V.V. (2018). PCM-mortar based construction materials for energy efficient buildings: A review on research trends. Energy Build..

[80] Berardi U., Gallardo A. (2019). Properties of concretes enhanced with phase change materials for building applications. Energy Build..

[81] Cellat K., Tezcan F., Kardaş G., Paksoy H. (2019). Comprehensive investigation of butyl stearate as a multifunctional smart concrete additive for energy-efficient buildings. Int. J. Energy Res..

[82] Cunha S., Leite P., Aguiar J. (2020). Characterization of innovative mortars with direct incorporation of phase change materials. J. Energy Storage.

[83] Parameshwaran R., Kumar G.N., Ram V.V. (2020). Experimental analysis of hybrid nanocomposite-phase change material embedded cement mortar for thermal energy storage. J. Build. Eng..

[84] Frigione M., Lettieri M., Sarcinella A., De Aguiar J.L.B. (2019). Applications of Sustainable Polymer-Based Phase Change Materials in Mortars Composed by Different Binders. Materials.

[85] Giro-Paloma J., Barreneche C., Maldonado-Alameda A., Royo M., Formosa J., Fernandez A.I., Chimenos J.M. (2019). Alkali-Activated Cements for TES Materials in Buildings’ Envelops Formulated With Glass Cullet Recycling Waste and Microencapsulated Phase Change Materials. Materials.

[86] Hekimoğlu G., Nas M., Ouikhalfan M., Sarı A., Kurbetci Ş., Tyagi V.V., Sharma R.K., Saleh T.A. (2020). Thermal management performance and mechanical properties of a novel cementitious composite containing fly ash/lauric acid-myristic acid as form-stable phase change material. Constr. Build. Mater..

[87] Hekimoğlu G., Nas M., Ouikhalfan M., Sarı A., Tyagi V.V., Sharma R.K., Kurbetci Ş., Saleh T.A. (2020). Silica fume/capric acid-stearic acid PCM included-cementitious composite for thermal controlling of buildings: Thermal energy storage and mechanical properties. Energy.

[88] Chang H., Jin L. (2020). Preparation and Heat Transfer Performance of Steel Ball Phase Change Concrete. J. New Mater. Electrochem. Syst..

[89] Shi J., Li M. (2021). Lightweight mortar with paraffin/expanded vermiculite-diatomite composite phase change materials: Development, characterization and year-round thermoregulation performance. Sol. Energy.

[90] Zhang X., Xu M., Liu L., Huan C., Zhao Y., Qi C., Song K.-I. (2019). Experimental study on thermal and mechanical properties of cemented paste backfill with phase change material. J. Mater. Res. Technol..

[91] Kheradmand M., Abdollahnejad Z., Pacheco-Torgal F. (2020). Alkali-activated cement-based binder mortars containing phase change materials (PCMs): Mechanical properties and cost analysis. Eur. J. Environ. Civ. Eng..

[92] Sarı A., Tyagi V.V. (2020). Thermal energy storage properties and lab-scale thermal performance in cementitious plaster of composite phase change material for energy efficiency of buildings. Environ. Prog. Sustain. Energy.

[93] Sarı A., Hekimoğlu G., Tyagi V., Sharma R. (2020). Evaluation of pumice for development of low-cost and energy-efficient composite phase change materials and lab-scale thermoregulation performances of its cementitious plasters. Energy.

[94] Valentini F., Morandini F., Bergamo M., Dorigato A. (2020). Development of eco-sustainable plasters with thermal energy storage capability. J. Appl. Phys..

[95] Hasanabadi S., Sadrameli S.M., Sami S. (2021). Preparation, characterization and thermal properties of surface-modified expanded perlite/paraffin as a form-stable phase change composite in concrete. J. Therm. Anal. Calorim..

[96] Illampas R., Rigopoulos I., Ioannou I. (2021). Influence of microencapsulated Phase Change Materials (PCMs) on the properties of polymer modified cementitious repair mortar. J. Build. Eng..

[97] Ramakrishnan S., Pasupathy K., Sanjayan J. (2021). Synthesis and properties of thermally enhanced aerated geopolymer concrete using form-stable phase change composite. J. Build. Eng..

[98] Shen Y., Liu S., Zeng C., Zhang Y., Li Y., Han X., Yang L., Yang X. (2021). Experimental thermal study of a new PCM-concrete thermal storage block (PCM-CTSB). Constr. Build. Mater..

[99] Wang M., Liu L., Zhang X.-Y., Chen L., Wang S.-Q., Jia Y.-H. (2019). Experimental and numerical investigations of heat transfer and phase change characteristics of cemented paste backfill with PCM. Appl. Therm. Eng..

[100] Dardir M., Panchabikesan K., Haghighat F., El Mankibi M., Yuan Y. (2019). Opportunities and challenges of PCM-to-air heat exchangers (PAHXs) for building free cooling applications—A comprehensive review. J. Energy Storage.

[101] De Falco M., Capocelli M., Losito G., Piemonte V. (2017). LCA perspective to assess the environmental impact of a novel PCM-based cold storage unit for the civil air conditioning. J. Clean. Prod..

[102] Kylili A., Fokaides P.A. (2016). Life Cycle Assessment (LCA) of Phase Change Materials (PCMs) for building applications: A review. J. Build. Eng..

[103] Thaib R., Hamdani H., Amin M. (2019). Utilization of Beeswax/Bentonite as energy storage material on building wall composite. J. Phys. Conf. Ser..

[104] Hakim I.I., Putra N., Agustin P.D. (2020). Measurement of PCM-concrete composites thermal properties for energy conservation in building material. AIP Conf. Proc..

[105] Ji R., Zou Z., Liu L., Wei S., Qu S. (2019). Development and energy evaluation of phase change material composite for building energy-saving. Int. J. Energy Res..

[106] Montanari C., Li Y., Chen H., Yan M., Berglund L.A. (2019). Transparent Wood for Thermal Energy Storage and Reversible Optical Transmittance. ACS Appl. Mater. Interfaces.

[107] Sarabandi D., Roudini G., Barahuie F. (2019). Activated carbon derived from pine cone as a framework for the preparation of n-heptadecane nanocomposite for thermal energy storage. J. Energy Storage.

[108] Liu Y., Yu K., Lu S., Wang C., Li X., Yang Y. (2020). Experimental research on an environment-friendly form-stable phase change material incorporating modified rice husk ash for thermal energy storage. J. Energy Storage.

[109] Temiz A., Hekimoğlu G., Köse Demirel G., Sarı A., Mohamad Amini M.H. (2020). Phase change material impregnated wood for passive thermal management of timber buildings. Int. J. Energy Res..

[110] Xie N., Niu J., Zhong Y., Gao X., Zhang Z., Fang Y. (2020). Development of polyurethane acrylate coated salt hydrate/diatomite form-stable phase change material with enhanced thermal stability for building energy storage. Constr. Build. Mater..

[111] Alassaad F., Touati K., Levacher D., Sebaibi N. (2021). Impact of phase change materials on lightened earth hygroscopic, thermal and mechanical properties. J. Build. Eng..

[112] Biesuz M., Valentini F., Bortolotti M., Zambotti A., Cestari F., Bruni A., Sglavo V.M., Sorarù G.D., Dorigato A., Pegoretti A. (2021). Biogenic architectures for green, cheap, and efficient thermal energy storage and management. Renew. Energy.

[113] Molinari C., Zanelli C., Laghi L., De Aloysio G., Santandrea M., Guarini G., Conte S., Dondi M. (2021). Effect of scale-up on the properties of PCM-impregnated tiles containing glass scraps. Case Stud. Constr. Mater..

[114] Rathore P.K.S., Kumar Shukla S. (2021). Improvement in thermal properties of PCM/Expanded vermiculite/expanded graphite shape stabilized composite PCM for building energy applications. Renew. Energy.

[115] Yousefi A., Tang W., Khavarian M., Fang C. (2021). Development of novel form-stable phase change material (PCM) composite using recycled expanded glass for thermal energy storage in cementitious composite. Renew. Energy.

[116] RAL Gütesicherung (2018). Phase Change Materials RAL-GZ 896.

[117] (2020). Standard Test Method for Using a Heat Flow Meter Apparatus for Measuring Thermal Storage Properties of Phase Change Materials and Products.

[118] International Energy Agency (IEA) (2020). Heat Pumps—Analysis.

[119] Vocale P., Morini G.L., Spiga M. (2014). Influence of Outdoor Air Conditions on the Air Source Heat Pumps Performance. Energy Procedia.

[120] Javadi H., Mousavi Ajarostaghi S.S., Rosen M.A., Pourfallah M. (2019). Performance of ground heat exchangers: A comprehensive review of recent advances. Energy.

[121] You T., Wu W., Shi W., Wang B., Li X. (2016). An overview of the problems and solutions of soil thermal imbalance of ground-coupled heat pumps in cold regions. Appl. Energy.

[122] Wu Y., Gan G., Gonzalez R.G., Verhoef A., Vidale P.L. (2011). Prediction of the thermal performance of horizontal-coupled ground-source heat exchangers. Int. J. Low-Carbon Technol..

[123] Habibi M., Hakkaki-Fard A. (2018). Evaluation and improvement of the thermal performance of different types of horizontal ground heat exchangers based on techno-economic analysis. Energy Convers. Manag..

[124] Dehdezi P.K., Hall M.R., Dawson A.R. (2012). Enhancement of Soil Thermo-Physical Properties Using Microencapsulated Phase Change Materials for Ground Source Heat Pump Applications. Appl. Mech. Mater..

[125] Bottarelli M., Bortoloni M., Su Y., Yousif C., Aydın A.A., Georgiev A. (2015). Numerical analysis of a novel ground heat exchanger coupled with phase change materials. Appl. Therm. Eng..

[126] Wang J.L., De Zhao J., Liu N. (2014). Numerical Simulation of Borehole Heat Transfer with Phase Change Material as Grout. Appl. Mech. Mater..

[127] Chen F., Mao J., Chen S., Li C., Hou P., Liao L. (2018). Efficiency analysis of utilizing phase change materials as grout for a vertical U-tube heat exchanger coupled ground source heat pump system. Appl. Therm. Eng..

[128] Qi D., Pu L., Sun F., Li Y. (2016). Numerical investigation on thermal performance of ground heat exchangers using phase change materials as grout for ground source heat pump system. Appl. Therm. Eng..

[129] Zhang M., Liu X., Biswas K., Warner J. (2019). A three-dimensional numerical investigation of a novel shallow bore ground heat exchanger integrated with phase change material. Appl. Therm. Eng..

[130] Bonamente E., Aquino A. (2020). Environmental Performance of Innovative Ground-Source Heat Pumps with PCM Energy Storage. Energies.

[131] Li X., Tong C., Duanmu L., Liu L. (2016). Research on U-tube Heat Exchanger with Shape-stabilized Phase Change Backfill Material. Procedia Eng..

[132] Yang W., Xu R., Yang B., Yang J. (2019). Experimental and numerical investigations on the thermal performance of a borehole ground heat exchanger with PCM backfill. Energy.

[133] Barbi S., Barbieri F., Marinelli S., Rimini B., Merchiori S., Larwa B., Bottarelli M., Montorsi M. (2021). Phase change material-sand mixtures for distributed latent heat thermal energy storage: Interaction and performance analysis. Renew. Energy.

[134] Zhu N., Hu P., Lei Y., Jiang Z., Lei F. (2015). Numerical study on ground source heat pump integrated with phase change material cooling storage system in office building. Appl. Therm. Eng..

[135] Pu L., Xu L., Zhang S., Li Y. (2019). Optimization of ground heat exchanger using microencapsulated phase change material slurry based on tree-shaped structure. Appl. Energy.

[136] Kong M., Alvarado J.L., Thies C., Morefield S., Marsh C.P. (2017). Field evaluation of microencapsulated phase change material slurry in ground source heat pump systems. Energy.

[137] Bonamente E., Aquino A., Cotana F. (2016). A PCM Thermal Storage for Ground-source Heat Pumps: Simulating the System Performance via CFD Approach. Energy Procedia.

[138] Bayomy A.M., Nguyen H.V., Wang J., Dworkin S.B. Performance analysis of a single underground thermal storage borehole using phase change material. Proceedings of the International Ground Source Heat Pump Association (IGSHPA) Research Track Conference.

[139] Chen F., Mao J., Li C., Hou P., Li Y., Xing Z., Chen S. (2018). Restoration performance and operation characteristics of a vertical U-tube ground source heat pump system with phase change grouts under different running modes. Appl. Therm. Eng..

[140] Faizal M., Bouazza A., Singh R.M. (2016). Heat transfer enhancement of geothermal energy piles. Renew. Sustain. Energy Rev..

[141] Mohamad Z., Fardoun F., Meftah F. (2021). A review on energy piles design, evaluation, and optimization. J. Clean. Prod..

[142] Han C., Yu X. (2018). An innovative energy pile technology to expand the viability of geothermal bridge deck snow melting for different United States regions: Computational assisted feasibility analyses. Renew. Energy.

[143] Mousa M.M., Bayomy A.M., Saghir M.Z. (2020). Experimental and Numerical Study on Energy Piles with Phase Change Materials. Energies.

[144] Sommerfeldt N., Madani H. (2019). In-depth techno-economic analysis of PV/Thermal plus ground source heat pump systems for multi-family houses in a heating dominated climate. Sol. Energy.

[145] Emmi G., Zarrella A., De Carli M. (2017). A heat pump coupled with photovoltaic thermal hybrid solar collectors: A case study of a multi-source energy system. Energy Convers. Manag..

